# A Nationwide Physical Activity Intervention for 654,500 Adults in Singapore: Cost-Utility Analysis

**DOI:** 10.2196/46178

**Published:** 2024-10-04

**Authors:** Gregory Ang, Chuen Seng Tan, Yot Teerawattananon, Falk Müller-Riemenschneider, Cynthia Chen

**Affiliations:** 1 Department of Statistics and Data Science, National University of Singapore, Singapore, Singapore; 2 Saw Swee Hock School of Public Health, National University of Singapore and National University Health System, Singapore, Singapore; 3 Yong Loo Lin School of Medicine, National University of Singapore, Singapore, Singapore; 4 Health Intervention and Technology Assessment Program, Ministry of Public Health, Nonthaburi, Thailand; 5 Digital Health Center, Berlin Institute of Health, Charité-Universitätsmedizin Berlin, Berlin, Germany; 6 Schaeffer Center for Health Policy and Economics, University of Southern California, Los Angeles, CA, United States; 7 Department of Non-Communicable Disease Epidemiology, The London School of Hygiene & Tropical Medicine, London, United Kingdom

**Keywords:** physical activity, mHealth, mobile health, nationwide program, Markov model, diabetes, hypertension, prevention, modeling study, productivity, cost, mortality, cost-effectiveness

## Abstract

**Background:**

Increasing physical inactivity is a primary risk factor for diabetes and hypertension, contributing to rising health care expenditure and productivity losses. As Singapore’s aging population grows, there is an increased disease burden on Singapore’s health systems. Large-scale physical activity interventions could potentially reduce the disease burden but face challenges with the uncertainty of long-term health impact and high implementation costs, hindering their adoption.

**Objective:**

We examined the cost-effectiveness of the Singapore National Steps Challenge (NSC), an annual nationwide mobile health (mHealth) intervention to increase physical activity, from both the health care provider perspective, which only considers the direct costs, and the societal perspective, which considers both the direct and indirect costs.

**Methods:**

We used a Markov model to assess the long-term impact of increased physical activity from the NSC on adults aged 17 years and older. A Monte Carlo simulation with 1000 samples was conducted to compare two situations: the NSC conducted yearly for 10 years against a no-intervention situation with no NSC. The model projected inpatient and outpatient costs and mortality arising from diabetes and hypertension, as well as their complications. Health outcomes were expressed in terms of the quality-adjusted life-years (QALYs) gained. All future costs and QALYs were discounted at 3% per annum. Sensitivity analyses were done to test the robustness of our model results.

**Results:**

We estimated that conducting the NSC yearly for 10 years with a mean cohort size of 654,500 participants was projected to prevent 6200 diabetes cases (95% credible interval 3700 to 9100), 10,500 hypertension cases (95% credible interval 6550 to 15,200), and 4930 deaths (95% credible interval 3260 to 6930). This led to a reduction in health care costs of SGD (Singapore dollar) 448 million (95% credible interval SGD 132 million to SGD 1.09 billion; SGD 1=US $0.73 for the year 2019). There would be 78,800 (95% credible interval 55,700 to 102,000) QALYs gained. Using a willingness-to-pay threshold of SGD 10,000 per QALY gained, the NSC would be cost-saving. When indirect costs were included, the NSC was estimated to reduce societal costs by SGD 1.41 billion (95% credible interval SGD 353 million to SGD 3.80 billion). The model was most sensitive to changes in the inpatient cost of treatment for diabetes complications, time horizon, and program compliance.

**Conclusions:**

In this modeling study, increasing physical activity by conducting a yearly nationwide physical activity intervention was cost-saving, preventing diabetes and hypertension and reducing mortality from these diseases. Our results provide important information for decision-making in countries that may consider introducing similar large-scale physical activity programs.

## Introduction

Physical inactivity is a primary risk factor for diabetes and hypertension [1]. Worldwide, hypertension is a major cause of premature mortality [2], while diabetes is expected to have caused 1.5 million deaths in 2019 [3]. Regular physical activity can potentially reduce the burden of these diseases, decreasing health care expenditure and productivity losses. Globally, physical inactivity is estimated to cost health care systems an equivalent to the purchasing power of US $53.8 billion annually, while in Singapore, the economic cost of physical inactivity is estimated to be equivalent to the purchasing power of US $201 million [4]. Yet, more than 20% of Singaporeans are physically inactive [5].

Increased physical activity is associated with lower incidence and mortality of certain diseases, such as diabetes and hypertension [6-9]. However, evidence from published physical activity randomized controlled trials (RCTs) is not enough to make informed decisions on the cost-effectiveness of physical activity interventions [10]. RCTs are often conducted on small, targeted samples (eg, high-risk groups) and in controlled settings, which do not mimic real-life circumstances [11]. Systematic reviews of mobile health (mHealth) RCTs also highlight the need for longer follow-ups to evaluate their interventions’ effectiveness [12,13].

The emergence of mHealth devices such as wearables has facilitated the collection of health-related data [14]. This has enabled more large-scale interventions on individuals’ physical activity behavior [15,16]. Furthermore, wearables, more specifically activity trackers, could increase individuals’ physical activity levels [17]. However, despite the many benefits of increased physical activity, conducting these large-scale interventions still poses significant challenges, including a sizable investment. As such, it would be justifiable to apply model-based evaluation to project the longer-term impact of a large-scale intervention using real-world evidence.

Cost-effectiveness studies enable policy makers to make informed choices by comparing the costs and benefits of public health interventions, prioritizing the allocation of health funds given resource constraints [18]. Although mHealth is often reputed to be cost-effective, there is limited evidence to back this claim [19]. Furthermore, only a few studies analyze the cost-effectiveness of scaled-up physical activity interventions using an app [15]. According to Rondina et al [15], the first cost-effectiveness study of a commercial physical activity app was only published in 2021. Given the limited evidence, more cost-effectiveness studies of these commercial physical activity apps, which have a longer follow-up period, are warranted.

The National Steps Challenge (NSC) was first introduced in 2015 by the Health Promotion Board to improve physical activity in Singapore. During each season, which is around 5 months long, participants earned HealthPoints according to their steps accumulated each day. The steps accumulated were objectively measured either using their own tracker or a free NSC tracker. These HealthPoints could then be exchanged for cash vouchers.

To date, six seasons of the NSC have taken place. Previous articles have provided detailed information on the NSC and evaluated the impact of the program on increasing engagement and physical activity but have not studied its impact on health outcomes [20-23]. This is the first study that evaluates the long-term cost-effectiveness of the NSC and its impact on health. In this study, we adopt a model-based cost-effectiveness analysis for the NSC. We developed the NSC Markov model, a computer simulation of diabetes, hypertension, and their complications in Singaporean adults aged 17 years and older. The model compared two situations: the NSC conducted yearly for 10 years against no NSC. Health outcomes were expressed in terms of the quality-adjusted life-years (QALYs) gained.

## Methods

### Markov Model

**Figure 1.**
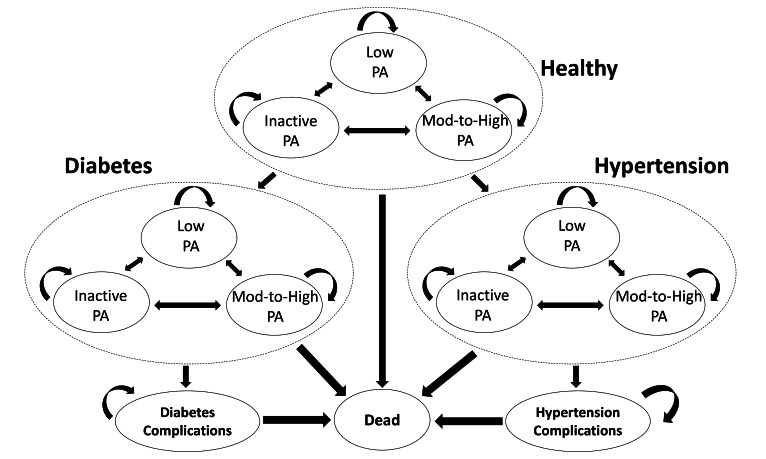
Structure of the National Steps Challenge (NSC) Markov model. Shown is the Markov model used to project costs, diabetes, hypertension, and quality-adjusted life-years of eligible adults for the NSC. The solid ovals denote the different states; the arrows denote possible transitions between states. Mod-to-High: moderate to high; PA: physical activity.

The NSC Markov model was constructed to estimate the costs and QALYs associated with diabetes, hypertension, and their complications in adults (Figure 1). We chose two diseases—diabetes and hypertension. The literature has shown physical activity has an impact in reducing the incidence and keeping these two diseases under control (see section 2 in Multimedia Appendix 1) [8]. The model first simulates a representative individual from the Singapore population. It then follows this individual, projecting the incidence of morbidity, death, and associated costs and health outcomes over 10 years. The model ran for 10 years, as this time horizon reflected a time period that decision-makers would find useful, given that the NSC has already been conducted for 6 years. The transition probabilities were specified using risk equations. There were three possible physical activity levels for each of the health states (healthy, diabetes and hypertension): inactive physical activity, low physical activity, and moderate to high physical activity. We modeled cardiovascular disease (CVD) as the main complication for diabetes and stroke as the main complication for hypertension. We took a conservative approach in estimating the benefits of the intervention by excluding comorbidity of diabetes and hypertension as well as other complications. Increased physical activity was assumed to decrease the risk of morbidities and the risk of mortality. The relative risks of diabetes, hypertension, and death for low and moderate to high physical activity compared to inactive physical activity were obtained from published literature [6-9].

The model compared two situations: the intervention situation had the NSC conducted yearly for 10 years against a no-intervention situation with no NSC. The cycle length was one year, with half-cycle corrections on costs and QALYs. We accounted for the health gained due to the increased physical activity from the NSC and for the payer’s direct costs of treatment for diabetes and hypertension by adopting the health system perspective. Societal costs were also estimated by adopting the societal perspective (Table S1 in Multimedia Appendix 1) [24,25]. All future costs and QALYs were discounted at 3% per annum [26].

Full details can be found in Multimedia Appendix 1. A presentation summary is also provided in Multimedia Appendix 2.

### Sample

We used data from the NSC to estimate the increase in physical activity for a closed cohort of Singapore residents aged 17 years and older. The age distribution of residents was obtained from the population census (Table S2 in Multimedia Appendix 1) [27].

The size of the NSC cohort was estimated to consist of 1.7 million unique participants registered across seasons 1 to 6 [28]. Also, a separate NSC study found that among 690,233 participants who signed up for NSC Season 3, 266,000 (38.5%) participants synced their trackers until the end of the challenge period [22]. Thus, using the same percentage of participants who continued to sync their trackers until the end of the NSC, we projected based on a closed cohort of 654,500 participants out of 1.7 million who had registered.

### Diseases and Mortality

Diabetes and hypertension prevalence rates (Figure S1 in Multimedia Appendix 1) and physical activity prevalence rates (Table B1 in Multimedia Appendix 1) were obtained from the 2010 National Health Survey [29]. The 2010 National Health Survey was chosen because we wanted the prevalence before NSC implementation in 2015. Diabetes and hypertension incidence rates, as well as their complications, were obtained from the Multi-Ethnic Cohort, a comprehensive study of 14,465 adults in Singapore (Figure S2, Table S1 in Multimedia Appendix 1) [30]. A multivariable probit regression was used to model the disease incidence, controlling for gender, race, educational attainment, age, BMI, marital status, and smoking. The transition probabilities from healthy to the disease for the different physical activity levels were computed using the disease incidence and their respective relative risk ratios (Table S1 in Multimedia Appendix 1) [6-9]. The mortality rates were extracted from the population census (Table S3 in Multimedia Appendix 1) [31]. The relative mortality risk ratios for the different physical activity levels and different diseases were obtained from relevant literature (Table S1 in Multimedia Appendix 1) [8,32,33].

### Costs and Utility Values of Health States

All costs were expressed in the Singapore dollar (SGD), where SGD 1=US $0.73=€0.66=CAD $0.98 for the year 2019 [34]. The direct costs of the diseases were computed using the inpatient and outpatient costs. The annual inpatient cost associated with treating diseases was obtained from the Ministry of Health using the median unsubsidized costs (Table S1 in Multimedia Appendix 1) [35]. The annual outpatient cost associated with treating diseases was obtained from relevant literature (Table S1 in Multimedia Appendix 1) [36,37]. For the diseases with no complications, the proportion of inpatient cases was also obtained from relevant literature (Table S1 in Multimedia Appendix 1) [36,38]. It was assumed that all diseases with complications were treated as inpatient cases. The total Health Promotion Board program and marketing expenses were SGD 120 million in 2018 [39]. Apart from the NSC, the Health Promotion Board conducts many national public health programs such as smoking cessation (IQuit program), eating healthily (My Healthy Plate), screening (Screen For Life), and vaccination programs for all ages, among others [40]. As the NSC reached out to 1.7 million participants, we applied a conservative approach, assuming that 30% of the Health Promotion Board budget was used for the NSC alone, with an annual cost of SGD 36 million. To incorporate the indirect costs of the diseases, the ratios of direct to indirect costs from a societal perspective were obtained from relevant literature (Table S1 in Multimedia Appendix 1) [24,25]. Health outcomes were evaluated based on utilities obtained for disease states and different physical activity levels (Table S1 in Multimedia Appendix 1) [6,41,42]. Utility values ranged from 0 (dead) to 1 (perfect health).

### Willingness-to-Pay Threshold

The Ministry of Health Agency for Care Effectiveness in Singapore did not provide a fixed willingness-to-pay threshold but a range of incremental cost-effectiveness ratios (ICERs) for the base case, with the lowest ICER at SGD 15,000 per QALY gained [26]. As the NSC is a physical activity intervention, the willingness-to-pay for preventive interventions might be lower than for medical interventions [43]. Hence, we used a lower willingness-to-pay threshold of SGD 10,000 per QALY gained.

### Estimation of Results

#### Overview of Analyses

In the previous subsections, we have described the parameters used for the base case. Scenario analysis, one-way deterministic sensitivity analysis, deterministic threshold sensitivity analysis, and probabilistic sensitivity analysis were done to assess the robustness of our model results in the base case to changes in key parameters over plausible ranges. All analyses were conducted in R version 4.1.2 (R Foundation for Statistical Computing).

#### Scenario Analysis

We examined the differentiation of cost among different physical activity levels within each health state. The base case assumed equal costs among different physical activity levels within each health state. As previous studies in the United States [44] and European Union [45] have found that higher physical activity levels reduced health care costs, we varied the costs for different physical activity levels within the same disease state, inflating the inactive physical activity states’ costs by 5% and deflating the costs for the moderate to high physical activity states by 5%. We assumed that physical activity helps to manage chronic diseases better, resulting in lower doctor visits and lower doses of medication, thus reducing medical costs by 5%.

#### Sensitivity Analyses

One-way deterministic sensitivity analysis was performed on 33 parameters by varying each parameter over their respective 95% confidence intervals (except inpatient and outpatient costs, program cost, program compliance, and time horizon), interquartile range (inpatient and ourpatient costs), and inflating (or deflating) the parameters by 30% (program cost, program compliance, and time horizon), while keeping the other parameters fixed (Table S1 in Multimedia Appendix 1). Deterministic threshold sensitivity analysis was performed on the three most sensitive parameters from the one-way deterministic sensitivity analysis.

Probabilistic sensitivity analysis was implemented by varying all 32 parameters (except time horizon) simultaneously using a Monte Carlo simulation with 1000 bootstrap samples, using prespecified distributions (Table S1 in Multimedia Appendix 1). The time horizon of 10 years was not varied, as this can be decided by the policy maker. Point estimates of cases averted, health care costs, QALYs gained, and the ICER (cost per QALY gained) were obtained by the mean of the 1000 bootstrap samples, and 95% credible intervals were obtained using the 2.5 and 97.5 percentiles. Lower willingness-to-pay thresholds of SGD 0 and SGD 5000 per QALY gained were also considered.

### Model Validation

The NSC Markov model was validated with real-world observations. The NSC Markov model’s projections of the 5-year physical activity prevalence rates were compared with observed physical activity levels from NSC Season 5 (Figure S4, Table B1 in Multimedia Appendix 1) [5]. The NSC Markov model’s projections of the 5-year disease prevalence rates were compared with data from the National Population Health Survey (2022) (Table B2 in Multimedia Appendix 1) [46].

### Ethical Considerations

Ethical approval for this study was obtained from the Institutional Review Board of the National University of Singapore (NUS-IRB LN-18-061E). Informed consent was obtained from all participants. The NSC data were deidentified. Participants were compensated according to the NSC rewards structure [23].

## Results

### Base Case

**Figure 2.**
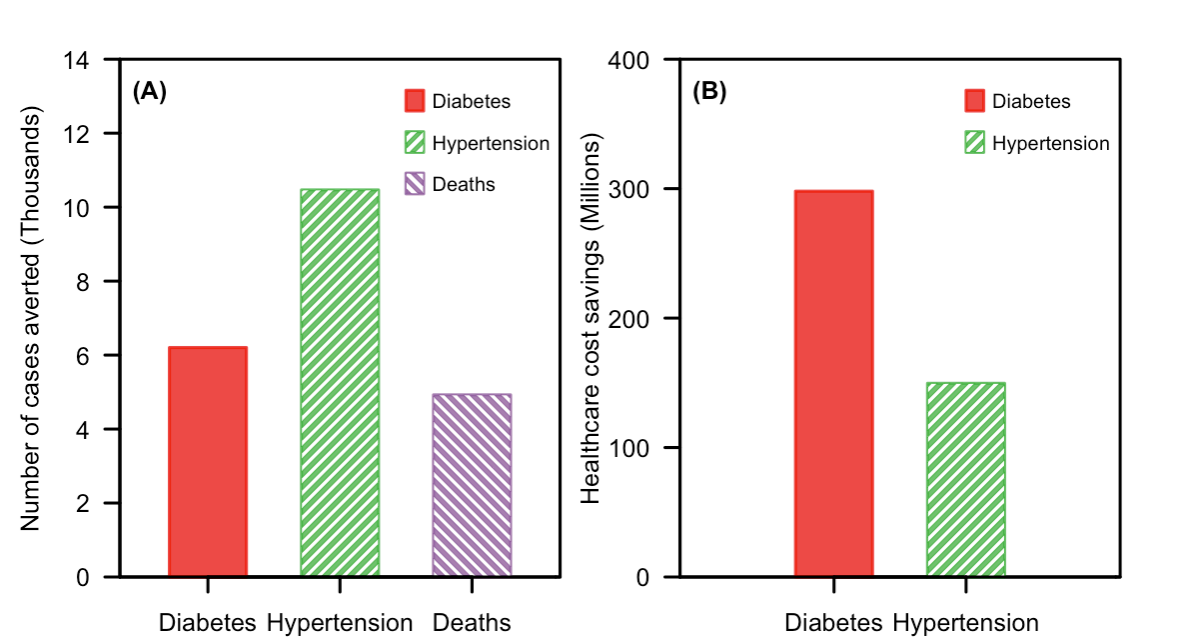
Projections from a health system perspective when the National Steps Challenge is conducted yearly for 10 years in the base case. Panel A shows the reductions in diabetes, hypertension, and deaths. Panel B shows the reduction in health care costs, which are expressed in Singapore dollars (SGD; SGD 1=US $0.73 for the year 2019).

Conducting the NSC yearly for 10 years with a mean cohort size of 654,500 participants aged 17 years and older was projected to prevent 6200 diabetes cases (95% credible interval 3700-9100), 10,500 hypertension cases (95% credible interval 6550-15,200), and 4930 death cases (95% credible interval 3260-6930) (Figure 2A), leading to 78,800 (95% credible interval 55,700-102,000) QALYs gained.

From the health system perspective, assuming no differentiation of cost among different physical activity levels within each health state, the health care cost savings from the averted cases was estimated to be SGD 448 million (95% credible interval SGD 132 million to SGD 1.09 billion), with SGD 298 million (95% credible interval SGD 34.7 million to SGD 925 million) for diabetes and SGD 150 million (95% credible interval SGD 46.5 million to SGD 328 million) for hypertension (Figure 2B). To achieve these savings, an investment of SGD 309 million (95% credible interval SGD 223 million to SGD 398 million) over 10 years for the program cost was required. Using a willingness to pay threshold of SGD 10,000, the NSC was cost-saving. From the societal perspective, the NSC was estimated to reduce societal costs by SGD 1.41 billion (95% credible interval SGD 353 million to SGD 3.80 billion) (Table 1) and was even more cost-saving.

**Figure 3.**
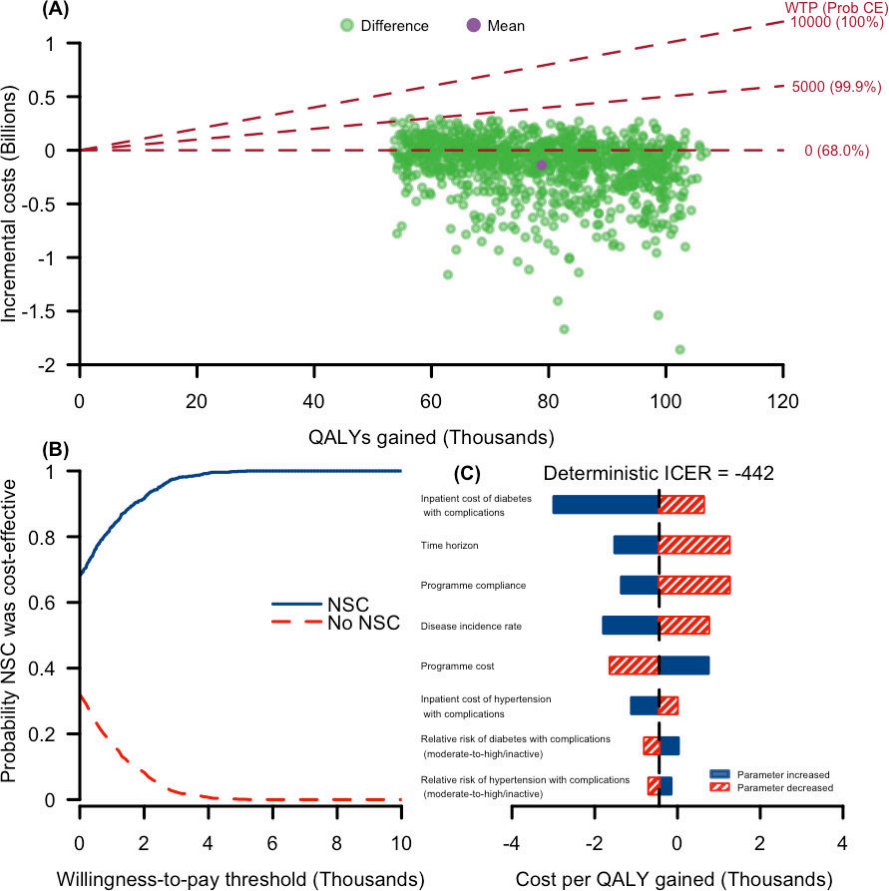
Results of probabilistic and one-way deterministic sensitivity analyses from a health system perspective when the NSC is conducted yearly over 10 years in the base case. Panel A shows the simulated incremental costs (in SGD; SGD 1=US $0.73 for the year 2019) and QALYs gained (green) from the probabilistic sensitivity analysis. The incremental cost is computed by subtracting the reduction in health care costs from the total program cost. The mean of the 1000 bootstrap samples was also plotted (purple). In each bootstrap sample, the NSC was cost-effective if the simulated point (green point) is below the willingness-to-pay threshold (red dotted lines). The percentages next to the willingness to pay are the proportion of bootstrap samples below the threshold, which estimates the probability that the NSC was cost-effective. Panel B shows the cost-effectiveness acceptability curve. With reference to panel A, the points (0, 0.680), (5000, 0.999), and (10,000, 1) lie on the blue curve in panel B. The remaining points on the blue curve in panel B were obtained by varying the willingness-to-pay threshold and computing the proportion of bootstrap samples below that threshold. The red curve is obtained by subtracting the proportion of bootstrap samples below the willingness-to-pay threshold (blue curve) from 1. Panel C shows the results of the one-way sensitivity analyses where model parameters were varied across a range of plausible values to see the impact on the cost per QALY gained. The deterministic ICER is obtained using the model parameters’ mean (or median for skewed parameters; eg, costs). The top 8 parameters (out of 33) to which the model was most sensitive are shown. Plausible ranges were preferentially derived from reported 95% confidence intervals or ranges or from calculated 95% confidence intervals, using standard errors as available, except for the inpatient rates, inpatient and outpatient treatment costs, program cost, program compliance, and time horizon. The interquartile range was used for the inpatient and outpatient treatment costs, and inflating/deflating the means by 30% was used for the inpatient rates, program cost, program compliance, and time horizon. ICER: incremental cost-effectiveness ratio; NSC: National Steps Challenge; QALY: quality-adjusted life-year; WTP (Prob CE): willingness-to-pay (probability that the NSC was cost-effective).

In the Monte Carlo simulations, all of the bootstrap samples (100%) had positive QALYs (Figure 3A), suggesting that the NSC improves health outcomes. From the health system perspective, there was a 68.0%, 99.9%, and 100% probability that the NSC was cost-effective at a willingness-to-pay of SGD 0, SGD 5000, and SGD 10,000 per QALY gained, respectively (Table 1). The cost-effectiveness of the NSC at increasing thresholds is shown in the cost-effectiveness curves (Figure 3B). This suggests that the NSC would be cost-effective at a willingness-to-pay threshold of SGD 10,000 per QALY gained. From the societal perspective (Table 1), there was a 97.7%, 100%, and 100% probability that the NSC was cost-effective at a willingness-to-pay of SGD 0, SGD 5000, and SGD 10,000 per QALY gained, respectively.

**Table 1.** Projected estimates of the cost and effectiveness if the NSC^a^ is conducted yearly for 10 years.^b^

Perspective^c^	Adjustment of costs due to PA^d^^,e^	Reduction in costs, in billions of SGD^f^^,g^ (95% credible interval)	ICER^h^ (costs per QALY^i^ gained), in thousands of SGD^j^	Cost-effectiveness (%)^k^		
				SGD 0 per QALY gained	SGD 5000 per QALY gained	SGD 10,000 per QALY gained
**Base case**						
Health system	Equal	0.448 (0.13-1.09)	Cost-saving^l^	68.0	99.9	100
Societal	Equal	1.41 (0.353-3.80)	Cost-saving^l^	97.7	100	100
**Scenario analysis**						
Health system	5%	0.518 (0.182-1.19)	Cost-saving^l^	82.0	100	100
Societal	5%	1.62 (0.481-3.99)	Cost-saving^l^	99.7	100	100

a NSC: National Steps Challenge.

b We assumed a 3% discount rate. For all the scenarios, including the base case, the simulated cohort size was 657,000 (469,000-844,000). The total program cost over 10 years was SGD 309 million (SGD 223 million to SGD 398 million). The QALYs gained were 78.8 thousand (55.7 thousand to 102 thousand). The brackets show the 95% credible intervals, which are obtained by the 2.5 and 97.5 percentiles of the 1000 bootstrap samples.

c Health system perspective only considers the direct cost of treatment of diseases. Societal perspective considers both the direct and indirect costs of treatment of diseases.

d For each disease state, we considered two settings. In the first setting, we assumed equal costs within the different physical activity levels (ie, equal). In the second setting, we used the costs for the low physical activity level as a benchmark and assumed a 5% lower cost for the moderate to high physical activity level and a 5% higher cost for the inactive physical activity level (ie, 5%).

e PA: physical activity.

f SGD: Singapore dollar (SGD 1=US $0.73 for the year 2019).

g The reduction in health care costs presented in this column does not take into account the total program cost over 10 years of SGD 309 million (95% credible interval SGD 223 million to SGD 398 million).

h ICER: incremental cost-effectiveness ratio.

i QALY: quality-adjusted life-year.

j The ICER (costs per QALY gained) was calculated by subtracting the reduction in health care costs from the total program cost and dividing the result by the QALYs gained.

k For each of the 1000 bootstrap samples, the ICER was computed. The probability that the NSC was cost-effective was calculated by computing the proportion of ICERs that were below the willingness-to-pay threshold (SGD 0, SGD 5000, or SGD 10,000 per QALY gained).

l The NSC was cost-saving if the QALY gained is positive and the reduction in health care costs exceeds the program cost. Hence, when the NSC is cost-saving, the ICER is negative.

### Scenario Analysis

From the health system perspective (Table 1), assuming a differential cost of 5% among participants with the same disease but with different physical activity levels, conducting the NSC yearly for 10 years with a mean cohort size of 654,500 participants was estimated to reduce health care costs by SGD 518 million (95% credible interval SGD 182 million to SGD 1.19 billion). After accounting for the program cost, the NSC was cost-saving (ie, improves health outcomes and the reduction of health care costs exceeds the program cost). There was a 82.0%, 100% and 100% probability that the NSC was cost-effective at a willingness-to-pay of SGD 0, SGD 5000, and SGD 10,000 per QALY gained, respectively. From the societal perspective (Table 1), there was a 99.7%, 100%, and 100% probability that the NSC was cost-effective at a willingness-to-pay of SGD 0, SGD 5000, and SGD 10,000 per QALY gained, respectively.

### One-Way Deterministic Sensitivity Analysis

From the health system perspective, the uncertainty ranges of individual parameters had an effect on the cost-effectiveness of the NSC (ICER range: cost-saving to SGD 1260 per QALY gained) (Figure 3C). The three parameters that the model was most sensitive to were (1) changes in the inpatient cost of treatment for diabetes with complications, (2) time horizon, and (3) program compliance. The model was also sensitive to changes in the disease incidence rate, the program costs, inpatient costs of treatment for hypertension with complications, and the relative risk of disease due to moderate to high physical activity compared to inactive physical activity for diseases with complications (diabetes and hypertension).

### Deterministic Threshold Sensitivity Analysis

From a health system perspective, the NSC was cost-saving if it is maintained in one of three ways: (1) program compliance was at least 34.7%, (2) the inpatient cost of treatment for diabetes complications was at least SGD 11,100, or (3) the NSC was conducted for at least 10 years.

## Discussion

### Principal Results

The NSC is an annual nationwide physical activity, having reached 1.7 million participants [28]. Based on a mean cohort size of 654,500 participants, we provide evidence of a cost-effective, scalable intervention with continuous objective activity monitoring of the individual’s physical activity behavior. We project that conducting the NSC yearly for 10 years improves health-related quality of life (as measured by QALYs) and reduces the number of diabetes and hypertension cases. The reduction in cases is estimated to save SGD 448 million in direct health care costs and an additional SGD 965 million when indirect costs such as productivity losses and costs due to early mortality are considered. The QALYs gained and cost savings are realized when the NSC is conducted annually, which delays the onset of diseases due to lower risk from higher physical activity levels.

An intervention is cost-effective if the cost per QALY gained is less than the willingness-to-pay threshold. Although the Ministry of Health Agency for Care Effectiveness in Singapore’s lowest willingness-to-pay threshold was SGD 15,000 per QALY gained, we chose a willingness-to-pay of SGD 10,000 per QALY gained as the willingness-to-pay threshold for preventive interventions might be lower than for medical interventions [43]. Other willingness-to-pay thresholds can also be considered in evaluating interventions. One common willingness-to-pay threshold is the gross domestic product (GDP) per capita (SGD 82,500 [47]), but this has its limitations [48]. For example, it does not consider if the intervention is affordable or feasible [48]. Another proposed willingness-to-pay threshold is SGD 30,500 per QALY gained, which is Singapore-specific and considers the opportunity costs of health care expenditure [49]. Future research could examine the appropriate willingness-to-pay thresholds for physical activity interventions. Nonetheless, as we set a lower threshold, using any of the aforementioned thresholds does not change our conclusion that the NSC is cost-saving.

A systematic review of 599 cost-effectiveness studies finds that the distributions of the cost-effectiveness ratios between treatment and preventive measures are similar [50]. While most preventive measures do not save money, they may still represent a reasonable investment and an efficient allocation of resources, as they provide health benefits at a low cost. Our results show that the NSC is a large-scale physical activity program that can deliver cost-effective prevention of noncommunicable diseases such as diabetes and hypertension. Also, with population aging, health care expenditure as a percentage of GDP has been rising, putting pressure on policy makers to keep health care affordable and focus on primary prevention [51]. In addition, the future program cost can be further reduced, as some existing infrastructure can be reused from earlier years, making the NSC more cost-saving. If health care costs rise faster than program costs and general inflation, preventive measures will be more cost-saving.

Our analysis showed a huge reduction in health care costs when we considered differential costs among participants with the same disease but with different physical activity levels (Table 1). This suggests that minimal change in clinical symptoms and costs for diabetes and hypertension can collectively contribute to a significant impact on public health as a whole. This also suggests that the reduction of health care costs is substantial if the diseases can be better managed by having a higher physical activity level. A study found that among people who had CVD, the average health care costs among those who met physical activity guidelines were more than SGD 3500 lower than those that did not meet guidelines [52]. Given that approximately one in five adults and one in two older adults live with more than one chronic condition, a large-scale physical activity intervention such as the NSC could be a cost-saving method to reduce the burden of chronic diseases [53].

One of the parameters that our study results were sensitive to was changes in program compliance (Figure 3C). The scalability and sustainability of a health care intervention are dependent on its acceptability [54]. A study of 132 reviews found that common measures of the acceptability of a health care intervention were attrition (studies: n=44, 33%) and compliance (studies: n=17, 13%) [54]. Better compliance with the intervention could enable participants to reap the benefits of the intervention. Nevertheless, the outreach of the NSC over the first five seasons is promising, reaching over 1.7 million unique participants (52%) in Singapore out of approximately 3.3 million residents aged 17 years and older [27,28]. Although NSC outreach is high and the program is currently cost-saving, our findings also suggest that the program could be even more cost-saving just by improving program compliance.

The NSC is a nationwide physical activity intervention. Conducting such an intervention has huge costs upfront due to the logistics and the cost of the incentives. However, these costs may eventually be offset due to the reduction in health care utilization due to a lower incidence of chronic diseases from increased physical activity. Since our time horizon is 10 years, we accounted for a plausible range of the differential costs and inflation in our cost-effectiveness analysis. At the same time, we estimate that the upfront financial costs associated with physical activity intervention are only partially mitigated by health care savings and acknowledge the risk that countries might face greater financial barriers due to the high implementation cost.

Even in the short term (five years), we project that there will be a decrease in the incidence of diabetes and hypertension cases and a decrease in mortality, leading to a reduction in health care costs and QALY gained (Figures S6A-C in Multimedia Appendix 1). Our results suggest that it would still be cost-effective to conduct the NSC in the short term (five years) regardless of whether the benefits of physical activity decreased across the year or persisted for the entire duration of the intervention (Figure S10 in Multimedia Appendix 1). However, the NSC was more cost-saving if conducted for a longer period. If the NSC was conducted throughout the participants’ lifetime, it could be even more cost-saving.

### Comparison With Prior Work

Our study results were similar to or more cost-effective than those of other cost-effectiveness studies on physical activity interventions (cost per QALY gained of these studies ranged from cost-saving to SGD 133,000) [15,55-66]. An instructor-led walking program, which also provided advice for inactive adults, had an ICER of SGD 133,000 per QALY gained [66]. When compared against other scaled-up physical activity interventions targeting individual behavior, the NSC was more cost-effective. Carrot Rewards, which used a commercial physical activity app, had a closed cohort sample of 38,452 participants (cost per QALY gained was SGD 10,900 over a five year time horizon) [15]. The building of urban greenways, another population-level physical activity intervention, was cost-effective (costs per QALY were SGD 6820 and SGD 28,100) [67]. A community-based physical activity intervention with 266 adults, “10,000 Steps Ghent,” was cost-saving, while another community-based physical activity intervention in the United Kingdom was cost-effective (cost per QALY was SGD 610) [65,68]. Furthermore, the cost-effectiveness of the NSC was similar to many of the RCTs on physical activity interventions included in systematic reviews [56,60,63,69]. The difference in cost-effectiveness of the NSC with other physical activity interventions can be attributed to the fact that the NSC includes all community-dwelling residents and is not focused on high-risk groups. As the incidence of diseases increases with age, the gains in disease reduction are greater among older adults [70,71]. More physical activity interventions targeted toward high-risk populations might be even more cost-effective. However, policy makers may find it difficult to deny interventions to certain groups of the population. Furthermore, it might also be more beneficial to encourage good physical activity behavior from a young age, influencing health in the long run.

### Limitations

Our analysis made several simplifying assumptions because of model and data limitations. First, as we conducted a closed cohort simulation, we did not consider additional participants who may have joined and benefited from future versions of the NSC. Second, we only modeled two diseases—diabetes and hypertension. There are other benefits from increased physical activity, such as reducing the risks of certain noncommunicable diseases, including lipid disorders (eg, high blood cholesterol) [72], dementia, and certain cancers [73]. Increased physical activity can reduce falls among older adults [74] and also reduce the incidence of the common cold [75]. Increased physical activity also has benefits for mental health, such as protecting against depression [76]. The reduction in the costs of treatment for these diseases and the long-term care costs are not accounted for in our model and would underestimate the NSC’s cost savings. Third, we assumed only one complication per disease and did not include a physical activity level in the complications states due to model complexity constraints and data availability. Fourth, we assumed that an adult would not contract both diabetes and hypertension. As patients with diabetes are likely to have hypertension, we assumed that as long as the adult had diabetes (regardless of whether they had hypertension), the adult would be classified under the diabetes state [77]. CVD is also a complication of both diabetes and hypertension. This would further underestimate the impact of the NSC. Fifth, increased physical activity is associated with increased worker productivity [78]. It may also be worthwhile for companies to introduce programs that promote physical activity in the workplace [78]. All these factors, which were not considered in this analysis, could make the NSC even more cost-saving.

The study has some methodological limitations. First, as the NSC was a nationwide program and not an RCT, our results do not establish a causal relationship between steps and morbidity or between steps and mortality. Second, selection bias may occur, as participation was voluntary. The NSC participants may be more health-conscious than the general population, leading to an overestimation of the effects of the NSC. Third, we assumed all participants with complications might use inpatient care; there might be an overestimation of the effects of the NSC. Fourth, within the NSC participants, due to the lack of data on noncompliant participants, we were unable to evaluate the difference between those who actively participated versus those who did not. Fifth, participants who were just diagnosed with diabetes, hypertension, or their complications may be more keen to participate in the NSC, whereas participants with advanced stages of complications may have lower levels of physical activity. We were unable to account for these behaviors in our analyses. Sixth, the expected cost of the NSC (SGD 309 million, SGD 36 million each year for 10 years at a 3% discount rate) may also be a limitation. With the anticipated increase in the number of participants, the variable costs associated with the NSC program are likely to rise, particularly due to the provision of participant incentives. Consequently, this may reduce the overall cost-effectiveness of the program. Budget constraints will influence the program’s impact, as larger rewards are associated with larger effects [79]. Furthermore, budget constraints will also determine the program’s level of outreach and engagement. However, the annual implementation of the NSC and our results that the NSC was cost-saving may indicate the sustainability of such a program, at least in high-income countries. This study also informs the long-term monitoring and evaluation (M&E) of physical activity programs in Singapore and should be applicable to other M&E plans for similar programs in other settings. However, we do not advocate the adaption of similar population-based programs without tailored M&E.

### Conclusions

In conclusion, this modeling study provides evidence of the cost-effectiveness of a nationwide physical activity intervention targeting individual behavior using an app. We projected that increased physical activity from a yearly nationwide physical activity intervention delayed the incidence of diabetes and hypertension and reduced mortality. With a conservative estimate of SGD 448 million in direct health care cost savings, our results suggest that this mHealth physical activity intervention is cost-saving and improves the quality of life. The estimated cost savings are more significant when indirect costs are considered. Hence, our results provide important information for decision-making in countries that may consider introducing similar physical activity programs.

## Supplementary material

10.2196/46178 Multimedia Appendix 1 Supplementary information on methodology, model parameters, and model results.

10.2196/46178 Multimedia Appendix 2 Presentation of the study.

## Abbreviations

CVD: cardiovascular disease
GDP: gross domestic product
ICER: increment cost-effectiveness ratio
M&E: monitoring and evaluation
mHealth: mobile health
NSC: National Steps Challenge
QALY: quality-adjusted life-year
RCT: randomized controlled trials
SGD: Singapore dollar
