# Supplementary material for: A Nationwide Physical Activity Intervention for 654,500 Adults in Singapore: Cost-Utility Analysis
Source: JMIR Public Health Surveill. 2024 Oct 4;10:e46178. doi: 10.2196/46178 (PMC11468974; doi:10.2196/46178)
Supplement: Multimedia Appendix 1 [file publichealth-v10-e46178-s001.doc]

# Supplementary Material

Table of Contents

Supplementary Methods (Pages 3 – 14)

1. Information on the National Steps Challenge^TM^
2. National Steps Challenge^TM^ Markov Model
3. Outcomes
4. Diseases
5. Death
6. Main Analysis
7. Effect of National Steps Challenge^TM^ on physical activity levels
8. Transition Probabilities
9. Sensitivity Analyses

Tables within the text (Pages 8 – 19)

(I) The NSC3 sample

- Table A1: Demographic information of the NSC3 sample

(II) Steps categories

- Table A2: Steps per day categories and classification.

(III) Computation of transition probabilities

- Table A3: Values from computing transition probabilities for diabetes.
- Table A4: Values from computing transition probabilities to diabetes from healthy.
- Table A5: Values from computing transition probabilities to diabetes complications from diabetes.

(IV) Time trending of physical activity prevalence

- Table A6**:** Prevalence rate of physical activity.

(V) Comparison

- Table B1**:** Prevalence rate of physical activity.
- Table B2: Prevalence rate of Diabetes and Hypertension by age group.

Supplementary Figures (Pages 15 – 24)

(I) Model Parameters

- Figure S1: Prevalence Rates of diabetes and hypertension.
- Figure S2: Incidence rates of diabetes, hypertension and their complications.
- Figure S3: Probability of death.

(II) Model Results

- Figure S4: Projected prevalence of physical activity for the no National Steps Challenge^TM^ (NSC) scenario and the NSC scenario over 10 years, based on a mean cohort size of 654,500 participants aged 17 and above.
- Figure S5: Projected prevalence of diseases for the no National Steps Challenge^TM^ (NSC) scenario and the NSC scenario over 10 years, based on a mean cohort size of 654,500 participants aged 17 and above.
- Figure S6: Cases averted and quality-adjusted life-years gained when the National Steps Challenge^TM^ (NSC) is conducted yearly over 10 years, based on a mean cohort size of 654,500 participants aged 17 and above for 5 and 10 years.
- Figure S7: Cumulative cases averted when the National Steps Challenge^TM^ (NSC) is conducted yearly over 10 years, based on a mean cohort size of 654,500 participants aged 17 and above.
- Figure S8: Quality-adjusted life-years gained when the National Steps Challenge^TM^ (NSC) is conducted yearly over 10 years, based on a mean cohort size of 654,500 participants aged 17 and above.
- Figure S9: Cumulative quality-adjusted life-years gained when the National Steps Challenge^TM^ (NSC) is conducted yearly over 10 years, based on a mean cohort size of 654,500 participants aged 17 and above.
- Figure S10: Comparison of the cost per quality-adjusted life-year gained over time between different decay rates when the National Steps Challenge^TM^ (NSC) is conducted yearly over 10 years, based on a mean cohort size of 654,500 participants aged 17 and above.
- Figure S11: Results of the probabilistic and one-way sensitivity analyses for the societal perspective when the National Steps Challenge^TM^ (NSC) is conducted yearly over 10 years, based on a mean cohort size of 654,500 participants aged 17 and above.

Supplementary Tables (Pages 25 – 34)

(I) Model Parameters

- Table S1: Base case parameters and ranges used in one-way and probabilistic sensitivity analyses.
- Table S2: Age distribution for Singapore Resident Population 2020.
- Table S3: Life table for Singapore Resident Population 2018.

(II) Model Results

- Table S4: Costs saved and quality-adjusted life-years gained based on probabilistic sensitivity analysis and deterministic analysis when the National Steps Challenge^TM^ (NSC) is conducted yearly over 10 years, with different initial physical activity prevalence rates, based on a mean cohort size of 654,500 participants aged 17 and above.
- Table S5: Costs saved due to differential costs from different physical activity levels due to better management of diseases using deterministic analysis when the National Steps Challenge^TM^ (NSC) is conducted yearly over 10 years, based on a cohort size of 654,500 participants aged 17 and above.
- Table S6: Costs saved and quality-adjusted life-years gained due to different discount rates using deterministic analysis when the National Steps Challenge^TM^ (NSC) is conducted yearly over 10 years, based on a cohort size of 654,500 participants aged 17 and above.

Supplementary References (Pages 35 – 37)

SUPPLEMENTARY METHODS

# 1. Information on the National Steps Challenge^TM^

The Singapore Health Promotion Board introduced the National Steps Challenge^TM^ (NSC) in 2015 to tackle the problem of physical inactivity. It aims to encourage Singapore residents to be more physically active every day, anytime and anywhere [1]. NSC promotes physical activity by using behavioural, economic, technological, environmental and gamification-related intervention components [2]. All Singapore residents aged 17 and above were eligible for NSC [1]. NSC participants were recruited via print posters, social media, and public roadshows.

During each NSC season, which is around five months long, participants earned HealthPoints according to their steps accumulated each day. The steps accumulated were objectively measured either using their own tracker or a free NSC tracker. The data was then synched with the Healthy365 mobile application.

The tiered system of rewards was intended to motivate participants to make even small but incremental changes to their physical activity habits. The HealthPoints accumulated could be exchanged for cash vouchers. Gain-framing was used (i.e., sure-win vouchers) to ensure that there would be few barriers to joining the intervention. For example, in NSC Season 3, achieving 5,000, 7,500 or 10,000 steps in a day earned 10, 25 or 40 HealthPoints, respectively. Participants start on Tier 1 and need to accumulate 5,250 HealthPoints to reach Tier 6 (the final tier). Every 750 HealthPoints earned participants $5, with a maximum of $35 upon the completion of all 6 tiers.

The main results of NSC have been published elsewhere [2-5]. More information on various seasons is also available in the NSC Frequently asked questions [1, 6].

# 2 National Steps Challenge^TM^ Markov Model

## 2.1 Cohort Size

We conducted a closed cohort Monte Carlo simulation. The age distribution of the participants was obtained from the Singapore resident population [7]. The model was populated with a hypothetical cohort with a mean of 654,500 resident participants [3].

654,500 resident participants were derived in the following manner. A separate NSC study found that among 690,233 participants who signed up for NSC Season 3, 266,000 (38.5%) participants synched their trackers until the end of the challenge period [2]. Thus, using the same percentage of participants who continued to sync their trackers till the end of NSC, we have made projections based on 654,500 participants out of 1.7 million who have registered for NSC [8].

## 2.2 Physical Activity levels

We obtained the daily step counts of the participants from NSC [3, 4]. Physical activity was classified into three different levels (inactive physical activity, low physical activity and moderate-to-high physical activity) based on weekly physical activity levels.

## 2.3 Disease States

We chose two diseases, diabetes and hypertension, that literature has shown that physical activity has an impact in reducing incidence and keeping it under control.

### 2.3.1 Diabetes

Diabetes is a chronic disease characterised by elevated levels of blood glucose (or blood sugar), which leads over time to complications involving the heart, blood vessels, eyes, kidneys and nerves [9]. The Ministry of Health Singapore in 2016 declared war on diabetes. Many health issues stem from diabetes. It is one of the diseases where an increase in physical activity would reduce the severity and incidence of the disease. About one in ten (9.5%) Singapore residents aged between 18 to 74 have diabetes while more than one in every five older adults aged 60 to 74 have diabetes [10]. We focused on type two diabetes, the most common form of diabetes, for our study. The main complication of diabetes that we will model is cardiovascular diseases (CVD), which accounted for 30.2% of deaths in Singapore in 2018 [11].

### 2.3.2 Hypertension

Apart from the war on diabetes, one of the other diseases afflicting Singapore is hypertension [12]. Hypertension or high blood pressure is a condition in which the blood vessels have persistently raised pressure [10]. Higher levels of physical activity are associated with a decreased risk of hypertension [13]. Over one in three (35.5%) Singapore residents aged between 18 to 74 have hypertension, while 74.9% of the older adults aged between 70 and 74 are hypertensive [10]. Hypertension is one of the key risk factors for stroke [10]. The main complication of hypertension that we modelled is stroke, which accounted for 6% of deaths in Singapore in 2018 [11].

## 2.4 The Model

We modelled three health states (a healthy state and two disease states, diabetes and hypertension) as these two diseases were of interest to us. Also, for each health state, we allowed for three different physical activity levels (inactive, low, and moderate-to-high). Similar to the assumption made by Vijay et al. [14], we assumed that the health states were mutually exclusive, and we did not allow the participant to become healthy after contracting a disease. In addition, we modelled CVD as the complication for diabetes and stroke as the complication for hypertension. Increased physical activity was assumed to decrease risk in morbidities and to decrease the risk of mortality. Thus, we had 11 “living” states and one “dead” state for a total of 12 states in the NSC Markov model.

# 3. Outcomes

## 3.1 Quality-adjusted life-year

To compute the quality-adjusted life-year (QALY), we would need to have a utility for each state. The utilities of the health states were obtained from Abdin et al [15]. The utilities are assumed to be the same across different cycles (i.e. time invariant). We computed the relative utilities of different physical activities for the healthy state and applied these relative utilities to the diabetes and hypertension states [14]. As the utility for diabetes with complications was not directly available, to compute the utility, we first computed the relative ratio of the utility for diabetes and at least one complication against the utility for diabetes and no complications [16]. We then multiplied this ratio with the utility of uncomplicated diabetes [15] to obtain the utility of diabetes with complications. The QALY at cycle *t* can be easily computed as follows:


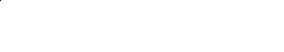


where *j* indexes the state,
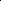
 is the utility for being in state *j* for one time period and
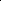
 is the proportion in state *j* at cycle *t*. We summed the weighted QALY (by discounting across the different cycles) to obtain the total present value of QALY at cycle 0. The different utilities associated with the different states are shown (**Table S1**).

From the literature search, we were unable to find utilities corresponding to physical activity levels and disease states; we were only able to obtain utility estimates for a specific disease state. As such, for each disease state, we set that estimate on the corresponding disease state with low physical activity level [14]. To obtain the utility for the inactive and moderate-to-high activity levels for the disease state, we used the relative proportions of the corresponding utilities in the Healthy state and multiplied it with the utility in the disease state for the low physical activity level.

## 3.2 Costs

### 3.2.1 Programme costs

We assumed that it cost SGD36 million per year to conduct the intervention. The total Health Promotion Board programme and marketing expenses were SGD120 million in 2018 [17]. Apart from NSC, the Health Promotion Board conducts many national public health programmes such as smoking cessation (I Quit programme), eating healthily (My Healthy Plate), screening (Screen For Life) and vaccination programmes for all ages, among others [18]. As NSC reached out to 1.7 million participants, we applied a conservative approach assuming that 30% of the HPB budget was used for NSC alone, with an annual cost of SGD36 million.

### 3.2.2 Treatment costs

The annual hospitalisation costs associated with the treatment of diseases were obtained from the Ministry of Health using the median unsubsidised costs based on private hospitals and clinics from 1 July 2018 to 30 June 2019 [19]. The annual outpatient costs associated with the treatment of diseases were obtained from relevant literature [20, 21]. For the diseases with no complications (diabetes and hypertension), the proportion of inpatient to outpatient cases was also obtained from relevant literature [10, 20]. It was assumed that all diseases with complications (diabetes and hypertension) were treated as inpatient cases. Costs were computed similarly as QALYs, replacing the utility with the cost incurred with being in that state.

As the base-case analysis, we assigned the same costs to the same disease, regardless of the physical activity levels. As an additional analysis, we set the costs to be that of the low physical activity level, inflated the costs for the inactive physical activity by 5% and deflated the costs for the moderate-to-high physical activity by 5%.

### 3.2.3 Societal Cost

As an additional analysis, we also considered the societal costs. A study estimated the indirect cost of diabetes is 2.8 times the direct cost of diabetes [22]. The indirect cost of diabetes accounted for (i) economic losses caused by workers missing work due to sickness (absenteeism), (ii) economic losses due to decreased worker productivity (presenteeism), (iii) non-participation in the labour force due to premature mortality and (iv) non-participation in the labour force due to diabetes [22]. For hypertension, a systematic review which included four countries found that on average 51.5% of total costs of hypertension were attributed to the direct costs [23]. As such, the indirect cost of hypertension is 0.94 times the direct cost of hypertension. The indirect costs of hypertension accounted for (i) presenteeism, (ii) absenteeism and (iii) non-participation in the labour force due to premature mortality [23]. The societal cost is the summation of the direct and indirect costs for diabetes and hypertension respectively.

# 4. Diseases

## 4.1 Initial Prevalence of Diseases

The crude prevalence rates for diabetes and hypertension were obtained from the 2010 National Health Survey [24]. As the model assumed that diabetes and hypertension were mutually exclusive, we subtracted the proportion of participants who had both diabetes and hypertension from the proportion of participants who had hypertension to avoid double-counting. However, as the prevalence rates differed significantly for each age, we smoothed the prevalence rates by fitting an isotonic regression to the prevalence rates (**Figure S1**). The isotonic regression ensured that the prevalence of both diseases is a non-decreasing function of age. The remaining proportion after subtracting the smoothed prevalence rates for diabetes and hypertension was assigned to “Healthy”.

We then extrapolated the smoothed prevalence for those aged 79 and above by using the smoothed prevalence from age 78. The initial probabilities for the states were obtained by multiplying the respective prevalence rates of the health state and physical activity level. Zero probability was assigned to both of the complication states and the death state at cycle 0.

## 4.2 Incidence rates of Diseases

The incidence rates of diabetes, hypertension and their complications were obtained from the Multi-Ethnic Cohort (MEC) [25]. A multivariable probit regression was used to model the 6-year incidence, controlling for gender, race, educational attainment, age, body mass index (BMI), marital status and smoking. The yearly incidence rates were then estimated based on five-year age groups (**Figure S2**) by assuming a constant incidence rate within the 6-year period (**Equation 1**).

The response variable was whether the participant contracted diabetes within the past 6 years (0: No, 1: Yes). The explanatory variables were gender, race, education attainment, 5-year age group, BMI, marital status and smoking. For the 5-year age groups, there were 8 indicator variables (45 to 49, …, 75 to 79, 80+), with the baseline age group set at 40 to 44. We fitted a probit model, and then estimated the mean diabetes incidence rate for age 40 to 44 by setting all the age indicator variables to be equal to 0, while the remaining explanatory variables are set to their mean values. We then estimated the mean diabetes incidence rate for age 45 to 49 by setting the 45 to 49 age indicator variable to be equal to be 1, the rest of the age indicator variables to be equal to 0, with the remaining explanatory variables are set to their mean values. This was repeated for the remaining age groups to estimate their respective mean diabetes incidence rates. The 95% confidence interval for the mean diabetes incidence rates were obtained via the delta method. Three other probit models were fitted to estimate the mean (and 95% confidence interval) of the incidence rates of hypertension, diabetes complication and hypertension complication.

As the inclusion criteria for the MEC cohort were age 40 and above, we had to extrapolate the disease incidence to those aged 17 to 39 in our hypothetical cohort. A linear regression[[1]](#footnote-2) was used, using a conservative estimate of the slope by visually inspecting the data points and using the data points where the linear trend seemed appropriate. The R-squared from these regressions were 0.964, 0.992, 0.898 and 0.990. From the extrapolation, some of the disease incidence of the youngest age groups were projected to be negative. These negative incidence values were then replaced with the last known positive incidence value from an older age group.

# 5. Death

We obtained the lifetable from the Department of Statistics in Singapore [26]. For age 17 to 99, it estimates the probability of dying at that age. We assumed this as the probability of dying under “Healthy” state, with the inactive physical activity level for the no-intervention situation with no NSC.

*Extension to above age 100*

As our analysis involved residents above age 100, we used a regression model to extrapolate the death probabilities. Our model was:


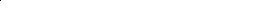


as we observed that taking the logarithm of both the death probabilities and age satisfied the linearity assumption requirement when compared to no logarithmic transformation of both the death probabilities and age.

We considered the death probabilities from those aged 70 and above. From our model, the maximum life expectancy was 115 (**Figure S3**).

# 6. Main Analysis

## 6.1 Base case

We used a typical Markov approach to time and model structure with a half-cycle correction [27]. The cycle length was one year since NSC was conducted annually, with half-cycle corrections on costs and QALYs. The time horizon was 10 years. Using the NSC Markov model, we projected the costs and QALYs under two different situations; the intervention situation had NSC conducted yearly for 10 years and the no-intervention situation had no NSC. Under the no-intervention situation, we assumed there was no change in the physical activity level [[2]](#footnote-3). For the intervention situation, we estimated the increase in physical activity based on the sample from NSC [4]. The costs and QALYs were reported on a present-value basis, with an annual discount rate of 3% [28].

## 6.2 Converting disease incidence to match cycle length of one year

The disease incidence rates were expressed in *n* = 6 years. We assumed that the disease incidence rates were constant within each 6-year period and took a “geometric mean” to obtain the disease incidence rates in yearly terms.

Hence, to obtain the yearly disease incidence rate:

|  | 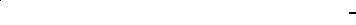 | (1) |
| --- | --- | --- |

# 7. Effect of NSC on physical activity levels

## 7.1 Initial Prevalence of physical activity

The initial prevalence of inactive, low and moderate-to-high physical activity was 39.1%, 40.5% and 20.4% [24].

## 7.2 Sample

The increase in physical activity in our NSC Markov model was estimated using data from NSC [4]. Our study involved participants from NSC3, which ran from 28 Oct 2017 to 31 Mar 2018. Participants were excluded from the current study if their demographics (sex, age) were missing, if their height and weight were missing or implausible (i.e., weight >300 kg, height >220 cm). Finally, as we needed to estimate the three-month transition probabilities for the physical activity levels, we only included participants who had at least one valid step count during the baseline period (28 Oct 2017 (start date of NSC3) – 3 Nov 2017) and at least one valid step count during the post-NSC period three months later (27 Jan 2018 – 2 Feb 2018). We obtained a closed cohort of 120,755 participants with mean (sd) age of 43.6 (13.6), mean (sd) BMI of 23.6 (4.2) with 38.7% male.

**Table A1** shows the number of participants and their mean age, BMI and percentage of males among the participants with (i) At least one valid step count during NSC3, (ii) at least one valid step count during the baseline period, (iii) at least one valid step count during the post-NSC period three months later and (iv) at least one valid step count during the baseline period and at least one valid step count during the post-NSC period three months later. Although there was a 35% decrease in the number of participants from (ii) to (iv), there were more participants in (iii) than (ii) due to the NSC Season 3 being a nationwide programme hence there were many participants who registered after one week from the start of NSC3. Furthermore, the mean ages and mean BMI for all four subsamples are similar, with only (iv) having a smaller percentage of males. Thus, the 120,755 participants estimate the intervention compliance well.

**Table A1:** Demographic Information of NSC3 sample

| **Condition** | **Number of participants** | **Mean Age (Sd)** | **Mean BMI (sd)** | **Percentage Male** |
| --- | --- | --- | --- | --- |
| At least one valid step count during NSC3 (28 Oct 2017 to 31 Mar 2018). | 395,428 | 41.6 (13.8) | 23.8 (4.47) | 41.3% |
| At least one valid step count during the baseline period (28 Oct 2017 (start date of NSC3) – 3 Nov 2017). | 184,457 | 42.3 (13.85) | 23.7 (4.33) | 40.1% |
| At least one valid step count during the post-NSC period three months later (27 Jan 2018 – 2 Feb 2018). | 231,866 | 43.0 (13.6) | 23.7 (4.29) | 40.0% |
| At least one valid step count during the baseline period (28 Oct 2017 (start date of NSC3) – 3 Nov 2017) and at least one valid step count during the post-NSC period three months later (27 Jan 2018 – 2 Feb 2018). | 120,755 | 43.6 (13.6) | 23.6 (4.2) | 38.7% |

## 7.2 Persistence of treatment effect in NSC situation

We made some key assumptions in order to extrapolate the increase in physical activity obtained from NSC. This can be done as the matrix product of 2 transition matrices is still a transition matrix.

We first constructed the three-month transition matrix Q_1_ using the NSC3 data. We averaged NSC3 participant’s step counts from 28 Oct 2017 – 3 Nov 2017 across the number of days recorded to determine the pre-physical activity level. The participant was then assigned one of the three physical activity levels (inactive physical activity, low physical activity or moderate-to-high physical activity) based on their average daily step count (**Table A2**) [29]. As we were interested in the effects on diseases due to moderate-to-high physical activity, we combined the two highest physical activity levels into one physical activity level.

**Table A2**: Steps per day categories and classification, modified from Cavero-Redondo, Tudor-Locke, et al [29]

| Steps per day | Physical Activity level |
| --- | --- |
| < 5000 | Inactive |
| 5000 – 7499 | Low |
| 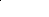 | Moderate-to-High |

The same procedure was repeated on the same participants three months later (27 Jan 2018 – 2 Feb 2018) to obtain their three month (post) physical activity level. The transition probabilities were then estimated based on the proportion of participants with the corresponding pre and post physical activity levels.

To construct the one-year transition matrix from the three-month transition matrix, we investigated different assumptions of decay on the NSC3 treatment effect: a) Full decay (base-case), b) No decay, c) 50% decay, 67% decay and 33% decay (**Figure S10**).

*a) Full decay (base-case)*

We used the transition matrix Q_1_ from the treatment situation for the first time period. Thereafter, we used the transition matrix computed from the no-treatment situation for the subsequent periods for the treatment situation.
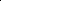
.

*b) No decay*

We used the same three-month transition matrix Q_1_ from the treatment situation for every time period.
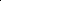
.

*c) 50%, 67% and 33% decay*

From our closed cohort, we were able to compute each participant’s
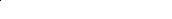
, the NSC3 treatment effect. The subscript 1 in the delta denotes the effect of the first quarter. We then allowed decay of the form
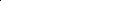
 where
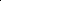
 denotes the time period in quarters and *k* controls the rate of decay. For a 33% decay, *k* = 0.33. Upon obtaining the decayed treatment effect, we then computed each participant’s estimated step counts in the post period and reobtained our transition matrix based on the proportion of participants with the corresponding pre and post physical activity levels. Hence in this case, the transition matrix for the treatment situation in each period changes.

From the different decay rates, we constructed the transition matrix from the 3^rd^ to 6^th^ month Q2, 6^th^ to 9^th^ month Q3 and 9^th^ to 12^th^ month Q4. We then multiplied these 4 matrices to obtain our one-year (one period) transition matrix.
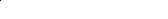
.

We regularized the impact of NSC by including a tuning parameter (
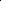
) in our model. The tuning parameter was included to enable the NSC Markov model to accurately reflect the impact of NSC on the entire Singapore resident population. Using the physical activity prevalence rates from the National Population Health Survey 2010 [24] and National Steps Challenge^TM^ Season 5 (NSC5), we set the increase in the moderate-to-high physical activity level from NSC5 to be the similar as the projected increase in the moderate-to-high physical activity level in our NSC Markov model for the first five years. After validating the model, the final transition matrix for the treatment situation (NSC) was: (
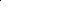
)


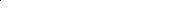


where
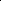
 is the three-by-three identity matrix which implies there was no change in physical activity levels. Since
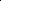
 and
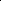
 are both transition matrices, and
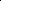
 is a convex combination of transition matrices, hence
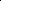
 is also a transition matrix.

## 7.3 Reduction of incidence rates for intervention situation

We assumed that an increased physical activity reduced the diabetes and hypertension incidence rate in the intervention situation. The reduction was computed based on the relative risks (**Table A3**) multiplied by the NSC transition matrix, which represents the weighted reduction in the incidence rate based on the proportion of participants in each of the three physical activity levels (**Equations 2, 3**). For example, in **Equation 2**, we assumed that NSC reduced the incidence of diabetes in the inactive physical activity level by a factor of (1-0.956) x 100% = 4.4%.

A step-by-step explanation of the computation of the reduction in the diabetes and diabetes complications incidence rates for the intervention situation (NSC) is described below. The computations were similar for hypertension.

**Table A3**: Values from computing transition probabilities for diabetes.

| Description | Value | Source |
| --- | --- | --- |
| Relative risk of diabetes for low physical activity compared to inactive physical activity | 0.93 | [14] |
| Relative risk of diabetes for moderate-to-high physical activity compared to inactive physical activity | 0.75 | [14] |
| Relative risk of diabetes complications for low physical activity compared to inactive physical activity | 0.86 | [30] |
| Relative risk of diabetes complications for moderate-to-high physical activity compared to inactive physical activity | 0.75 | [30] |

Assuming a 100% decay,


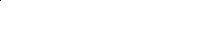


|  | 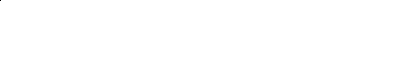 | (2) |
| --- | --- | --- |
|  |  |  |
|  | 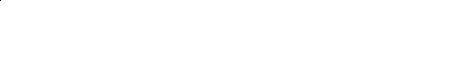 | (3) |

## 7.4 Transition matrix for the no-intervention situation

Since we assumed there was no change in physical activity in the no-intervention situation, the final transition matrix for the no-intervention situation was:


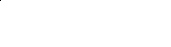


i.e.
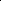
 is the three-by-three identity matrix.

# 8. Transition Probabilities

We defined the transition probabilities for the two different situations, where there was no NSC (no-intervention situation) and where NSC was conducted yearly for 10 years (intervention situation).

## 8.1 Explanation of Zero Probabilities

### 8.1.1 Dead state

Once the participant was dead, they stayed dead.

### 8.1.2 No recovering from diabetes or hypertension

In our model, we did not allow the participant to become healthy once they had contracted diabetes or hypertension.

### 8.1.3 No recovering from complications

Once the participant contracted a complication, they did not recover. They either stayed in that complications state, or they died. An assumption of the Markov model was that states are mutually exclusive. We did not allow a participant to contract both diabetes and hypertension. In reality, many participants who had diabetes also have hypertension. We assigned these participants in our model to the diabetes state as diabetes is more costly than hypertension.

### 8.1.4 Transiting from Healthy to Complications state

We did not allow Healthy to transition to complications in one cycle. The participant would either contract diabetes or hypertension in one cycle first, and then get complications in another cycle.

## 8.2 Death Probabilities

### 8.2.1 Adjusting Death Rates by Disease State

To obtain the probability of dying under diabetes, hypertension, diabetes complication (CVD) and hypertension complication (Stroke), we multiplied the death probability [26] with the respective diabetes, hypertension, diabetes complication or hypertension complication relative risk ratio [31, 32].

### 8.2.2 Adjusting Death Rates by Physical Activity levels.

To obtain the adjusted probability of dying for the different physical activity levels, we multiplied the relative risks of mortality for low and moderate-to-high physical activity levels [30].

pdeath(health_state = *i*, PA_level = *j* )

:= P(health_state_t+1_ = Dead | health_state_t_ = *i*, PA_level_t_ = *j*)

= P(health_state_t+1_ = Dead | health_state_t_ = *i*)
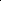
 (relative_risk for Inactive to *j* for health_state_t_ = *i*)

where *i* is either Healthy, Diabetes or Hypertension, *j* is either Inactive, Low or Moderate-to-High physical activity.

## 8.3 Probability of transitioning from Healthy to Diabetes/Hypertension

The probability that the participant transitions from Healthy to either Diabetes, Hypertension or their complications is the incidence rate of the respective disease which was elaborated in Section 4.2.

### 8.3.1 Transitioning from Healthy and Inactive Physical Activity

The diabetes (hypertension) incidence rate is the sum of the probabilities of moving from Healthy to any of the three physical activity levels in diabetes (hypertension). For the no-intervention situation, the three transition probabilities from (Healthy and Inactive) to (Diabetes and x), where x = Inactive, Low or Moderate-to-High were computed such that they summed to the diabetes incidence rate. For the intervention situation, we reduced the incidence rates for different physical activity levels according to the rates computed in section 7.3.

We illustrate step by step how the diabetes incidence probability for a theoretical participant aged 40 was computed. The transition probabilities for hypertension were computed in the same manner.

**Table A4**: Values from computing transition probabilities to diabetes from healthy.

| Description | Value | Source |
| --- | --- | --- |
| Transition probability to diabetes from healthy at age 40 | 0.012* | [25] |
| Relative risk of diabetes for low physical activity compared to inactive physical activity | 0.93 | [14] |
| Relative risk of diabetes for moderate-to-high physical activity compared to inactive physical activity | 0.75 | [14] |

*The one-year value were computed using Equation 1.

*No-* *intervention situation:*

P(
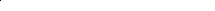
|
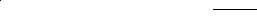


P(
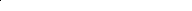
|
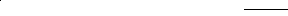


P(
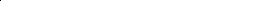
|
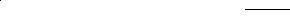


The probabilities were computed such that they summed to the incidence rate.

*Intervention situation:*

P(
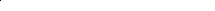
|
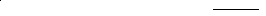
 0.956

P(
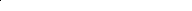
|
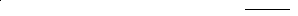
 0.902

P(
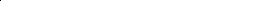
|
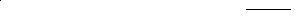
 0.759

where the additional terms were computed from Equation 2.

### 8.3.2 Adjusting Transition Probabilities from Healthy by Physical Activity levels

The steps from the previous section assumed that the participant had an inactive physical activity level before transition. We outline step by step how the diabetes incidence probabilities for a theoretical participant aged 40 with low or moderate-to-high physical activity were computed. The transition probabilities for hypertension were computed in the same manner.

*No-intervention situation:*

- *Low Physical Activity*

P(
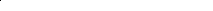
|
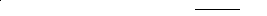


P(
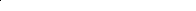
|
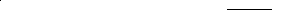


P(
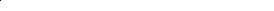
|
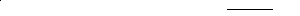


- *Moderate-to-High Physical Activity*

P(
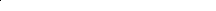
|
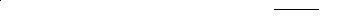


P(
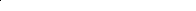
|
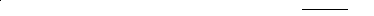


P(
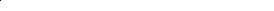
|
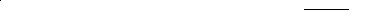


*Intervention situation:*

- *Low Physical Activity*

P(
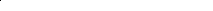
|
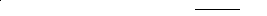

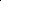


P(
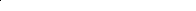
|
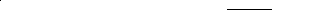


P(
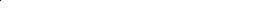
|
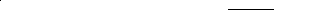


- *Moderate-to-High Physical Activity*

P(
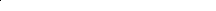
|
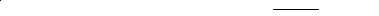


P(
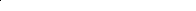
|
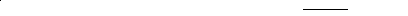


P(
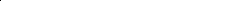
|
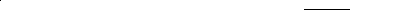


where the additional terms were computed from Equation 2.

## 8.4 Probability of transitioning to complications state

### 8.4.1 From complications

Given that the participant had complications, he or she either remained in the same health state or died. Since we had the death probability and probabilities must sum to one, the probability of staying in the complications state was one minus the death probability.

### 8.4.2 From no complications

Since we did not allow the participant to transition from Healthy to complications in one cycle, the probability of transitioning from no complications to the complications state is just the respective disease complication’s incidence rate. We again illustrate step by step the computations of diabetes complications for a theoretical participant aged 40. The computations for hypertension complications were similar. Recall that there were no physical activity levels in the complication states.

**Table A5**: Values from computing transition probabilities to diabetes complications from diabetes.

| Description | Value | Source |
| --- | --- | --- |
| Transition probability to diabetes complications from diabetes at age 40 | 0.013* | [25] |
| Relative risk of diabetes complications for low physical activity compared to inactive physical activity | 0.89 | [33] |
| Relative risk of diabetes complications for moderate-to-high physical activity compared to inactive physical activity | 0.79 | [33] |

*The one-year values were computed using Equation 1.

*No-intervention situation:*

P(
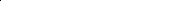
|
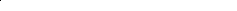


P(
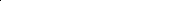
|
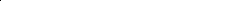


P(
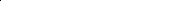
|
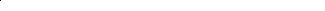


*Intervention situation:*

P(
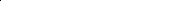
|
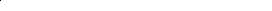


P(
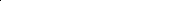
|
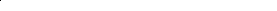


P(
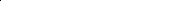
|
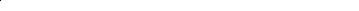


where the additional terms were computed from Equation 3.

## 8.5 Probability of staying in the same health state

The rows must sum to one. We subtract all the remaining probabilities in the same row from 1 to obtain the probability of no change in health state. To distribute the probabilities among the three different physical activity levels, we use
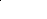
 for the no-intervention situation and
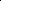
 for the intervention situation.

# 9 Sensitivity Analyses

## 9.1 One-way sensitivity analysis

One-way sensitivity analysis was performed on 33 parameters over their respective 95% confidence intervals (except inpatient and outpatient treatment costs, programme cost, programme compliance and time horizon), interquartile range (inpatient and outpatient treatment costs) and inflating (or deflating) the parameters by 30% (programme cost, programme compliance and time horizon) (**Table S1**). This was done by (i) computing the ICER using the mean values of other 32 parameters and the lower bound of the parameter of interest, then (ii) repeated for the upper bound of the parameter of interest and repeated for the other 32 parameters. Steps (i) and (ii) were repeated for each of the 33 parameters. The 33 parameters were then sorted in decreasing order in terms of the absolute change in ICER and the 8 parameters with the greatest absolute change in ICER plotted in a tornado plot. One-way sensitivity analysis was done from both the healthcare provider perspective (direct costs only) and the societal perspective (direct and indirect costs combined) (**Figure S11**).

## 9.2 Probabilistic Sensitivity Analysis

Probabilistic analyses examined the joint distribution of the parameters in the model via simulations, by generating 1000 random draws from pre-specified distributions (**Table S1**). Inpatient treatment costs and relative risks were modelled using a Gamma distribution, where the parameters were estimated using percentile matching using the R package ‘rriskDistributions’ [34]. Outpatient treatment costs were modelled using a Gamma distribution, where the parameters were estimated using the method of moments. Utilities were modelled using a beta distribution, where the parameters were estimated using the method of moments. The disease incidence was modelled using a beta distribution, where the parameters were estimated using the method of moments. We used these results to determine the uncertainty of the cost and effectiveness estimates using the 2.5 and 97.5 percentiles as an estimate of the 95% credible interval. SGD10,000 was used as the willingness-to-pay threshold.

## 9.3 Scenario Analysis

We conducted a deterministic analysis using the mean (or median for skewed parameters, e.g., costs) of the model parameters (**Table S4**). In the United States, the estimate of the physical inactivity prevalence ranged from 17.3% to 47.7% [35]. Apart from the base-case, we considered three different initial physical activity prevalence rates, (i) a scenario with low levels of physical activity (inactive: 50%, low: 25%, moderate-to-high: 25%), (ii) a scenario with high levels of physical activity (inactive: 20%, low: 40%, moderate-to-high: 40%) and (iii) a scenario using predicted physical activity levels in 2013 (described in the next paragraph) (**Table S4**). For the reduction in healthcare costs, apart from the 0% (base-case) and 5% differential costs that were considered, we also considered a low differential cost of 2.5% and high differential cost of 10% (**Table S5**). We also considered a discount rate of 0%, 5% and 10% on the costs and QALYs (**Table S6**).

**Table A6:** Prevalence rates of physical activity

| Data | Inactive | Low | Moderate-to-High | Source |
| --- | --- | --- | --- | --- |
| NHSS2007 | 17.7% | 43.2% | 39.1% | [36] |
| NHS2010 | 39.1% | 40.5% | 20.4% | [24] |

Since the physical activity prevalence rates were obtained from a 2010 National Health Survey 5 years ahead of NSC implementation in 2015, we also considered a time trend in the physical activity prevalence rates using the 2007 National Health Surveillance Survey (**Table A6**). We used relative change to trend the prevalence rates.

- *Moderate-to-High Physical Activity:* (1-(39.1-20.4)/39.1) × 20.4 = 10.64%
- *Low Physical Activity:* (1-(43.2-40.5)/43.2) × 40.5 = 37.97%
- *Inactive:* since percentages are bounded, it will be 100-37.97-10.64 = 51.39%

No time trending for the disease prevalence rates was conducted as we only had age-specific disease prevalence in 2010.

**Table S4** shows the results by varying the physical activity prevalence. By considering the time trend in the physical activity prevalence rates, there was an increase in healthcare cost savings and quality-adjusted life years, leading to an increase in the cost per QALY. However, these changes were minimal and did not change the conclusion that NSC is cost-saving.

NSC is more cost-effective when there are larger differential costs from different physical activity levels due to better management of diseases (**Table S5**) and when the discount rate is low (**Table S6**).

SUPPLEMENTARY FIGURES

**(I) Model Parameters**

**Figure S1:** Prevalence rates of diabetes and hypertension [24].


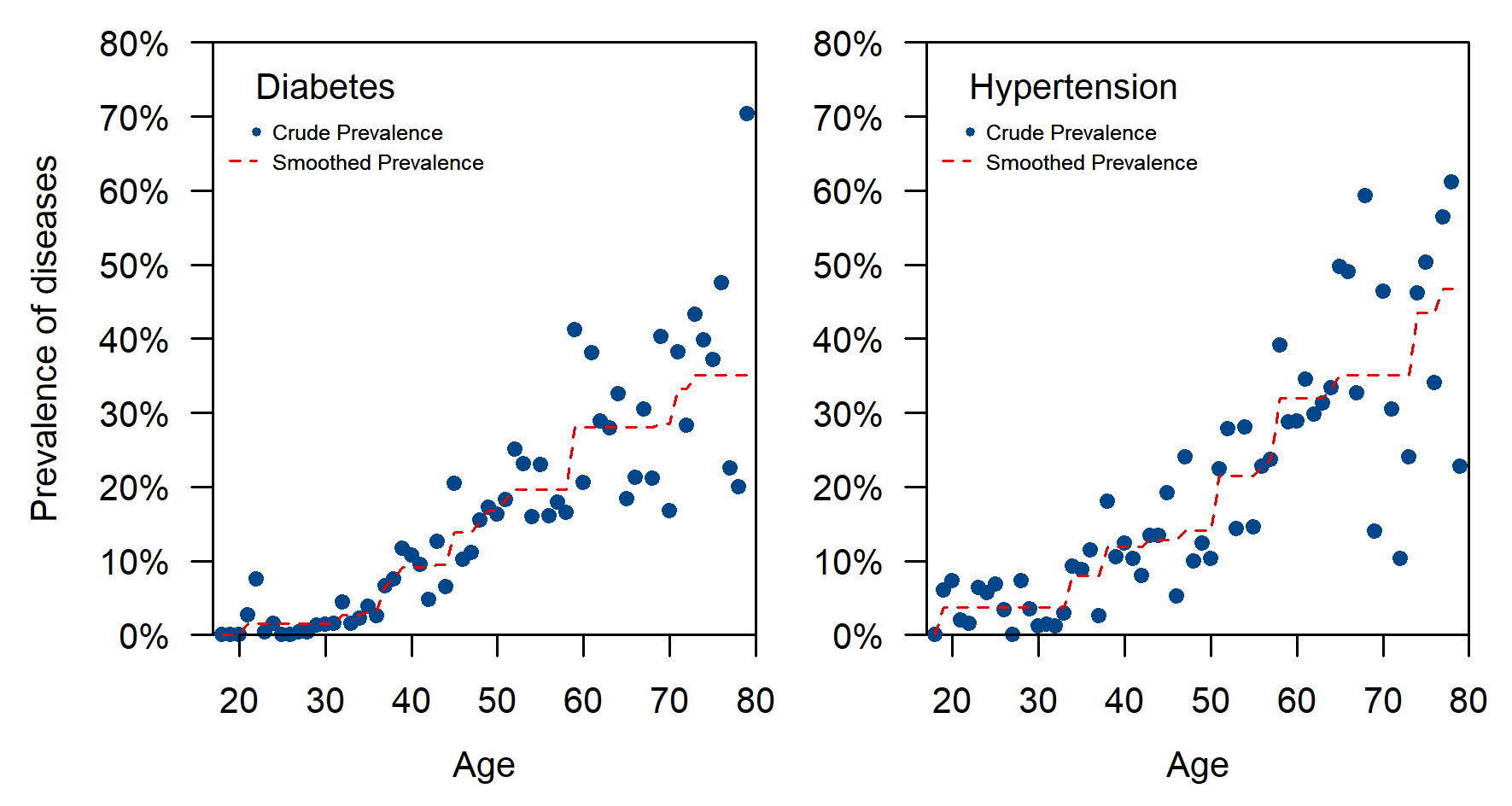


The figure shows the results of the smoothed prevalence rates for diabetes and hypertension. An isotonic regression was fitted to the data. The crude prevalence is obtained by taking the proportion of the sample which had the disease at that age. The smoothed prevalence was extrapolated for those aged 79 and above by using the smoothed prevalence from age 78.

**Figure S2:** Incidence rates of diabetes, hypertension and their complications [25].


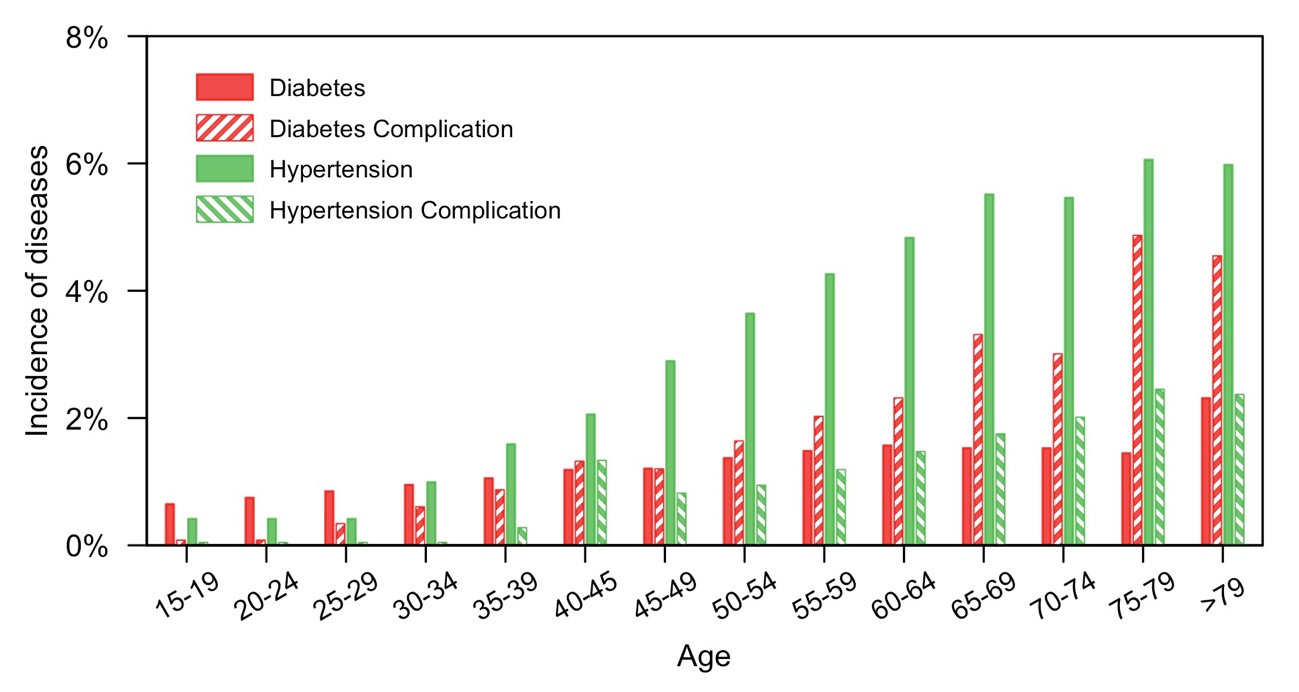


The figure shows the incidence rate of diabetes, hypertension and their complications obtained from the Multi-Ethnic Cohort.

**Figure S3:** Death Probability [26].


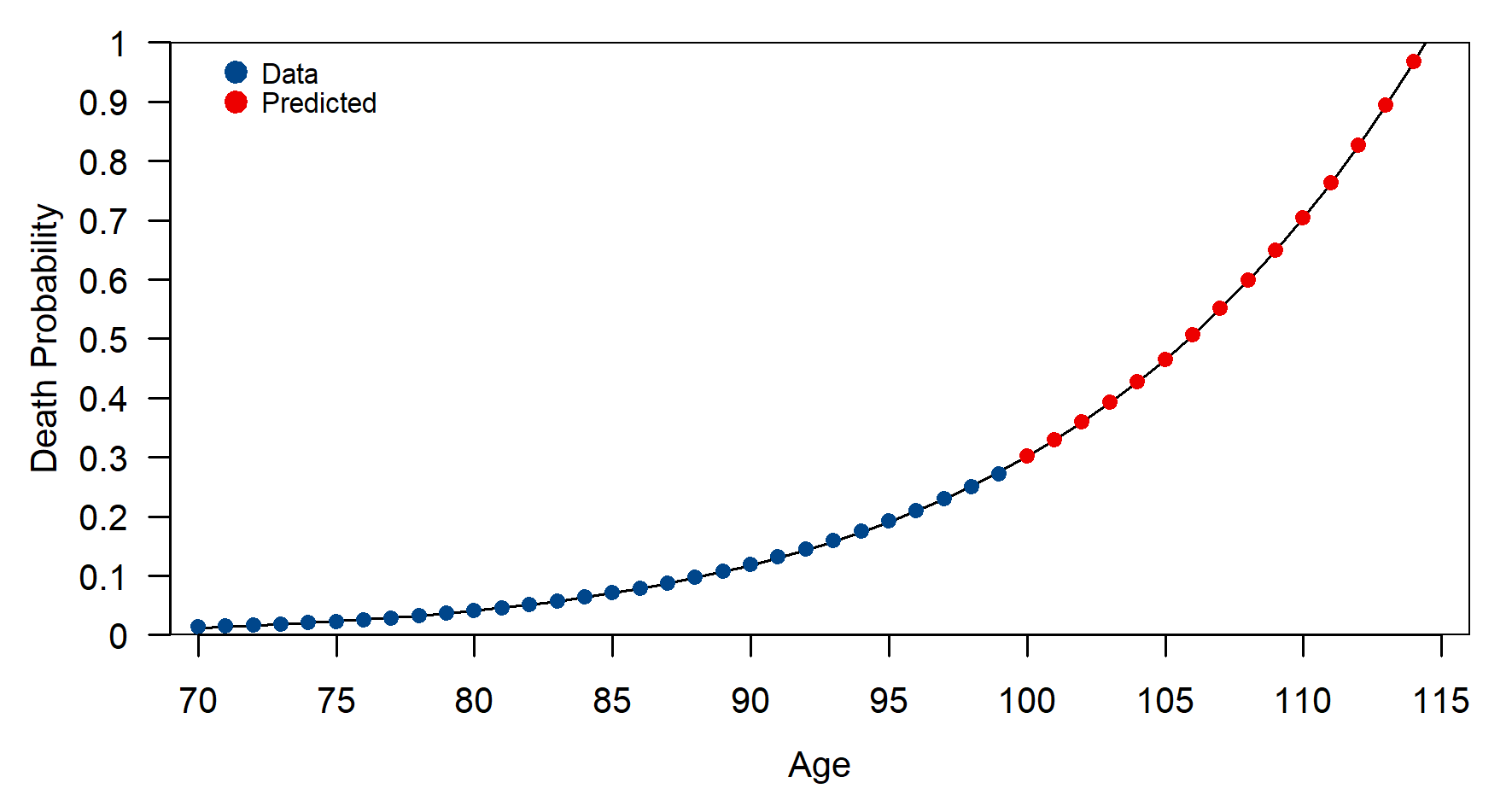


The figure shows the projected death probabilities for participants aged 100 and above. The model

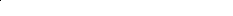
 was fitted to the data.

**(II) Model Validation**

**Figure S4:** Projected prevalence of physical activity for the no National Steps Challenge^TM^ (NSC) scenario and the NSC scenario over 10 years, based on a mean cohort size of 654,500 participants aged 17 and above.


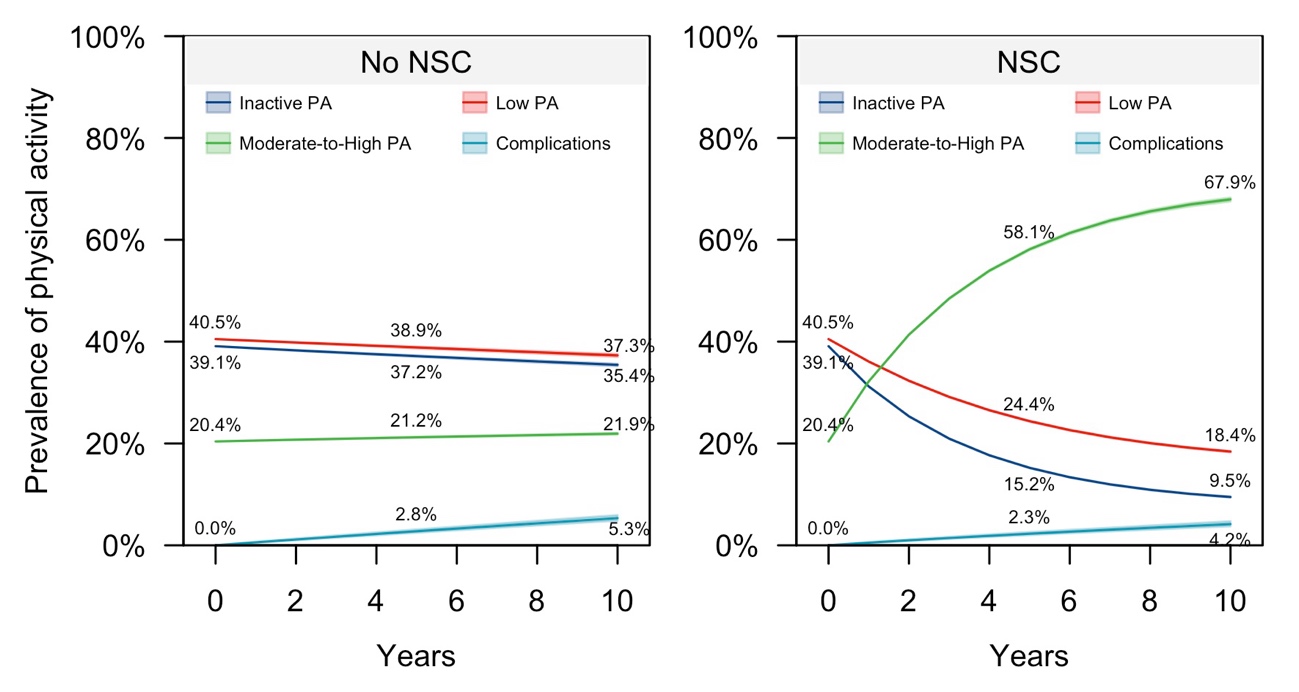


The solid lines represent the mean and the shaded regions represent the 95% credible intervals which are obtained using the 2.5 and 97.5 percentiles of the 1000 bootstrap samples.

As we did not model the physical activity level for diabetes complications and hypertension complications (eg. stroke, amputation) separately, they are grouped together as a group called “Complications”.

PA – Physical Activity; NSC – National Steps Challenge^TM^

**Table B1:** Prevalence rates of physical activity

| Data | Inactive | Low | Moderate-to-High | Source |
| --- | --- | --- | --- | --- |
| NHS2010 | 39.1% | 40.5% | 20.4% | [24] |
| NSC3^a^ | 16.1% | 25.3% | 58.6% | Using the respective datasets and classifying the physical activity levels using **Table A2**. |
| NSC5^a^ | 15.8% | 27.3% | 56.9% |

**Table B1** shows the prevalence of physical activity levels based on NHS2010 [24], NSC3 and NSC5, where each row sums to 100%. Both intervention and no-intervention situations (NSC and no NSC) have the same prevalence at year 0 based on the prevalence of physical activity from National Health Survey 2010 [24] (**Table B1**). At year 5, the projections for the intervention situation (NSC) based on our model (**Figure S4**) are similar to the prevalence rates obtained from NSC5 (**Table B1**), hence validating the model. The projections for the no-intervention situation (no NSC) were obtained by assuming no change in physical activity but allowing participants to develop complications.

**Figure S5:** Projected prevalence of diseases for the no National Steps Challenge^TM^ (NSC) scenario and the NSC scenario over 10 years, based on a mean cohort size of 654,500 participants aged 17 and above.


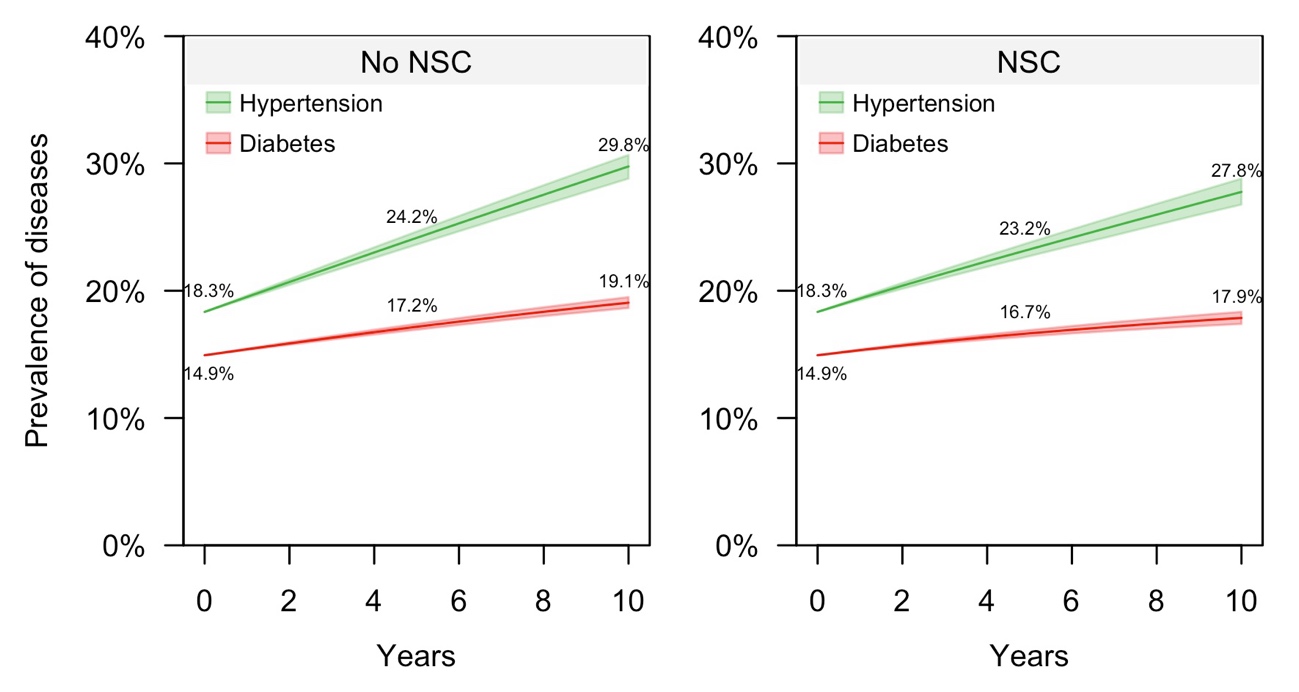


The solid lines represent the mean and the shaded regions represent the 95% credible intervals which are obtained using the 2.5 and 97.5 percentiles of the 1000 bootstrap samples.

NSC – National Steps Challenge^TM^

Both intervention and no-intervention situations (NSC and no NSC) have the same disease prevalence at year 0 based on the smoothed disease prevalence from the National Health Survey 2010 (**Figure S1**). As this is a cohort simulation, the disease prevalence rates are driven by the ageing effect, thus the overall percentages between the two scenarios may seem similar. However, even a small difference in percentages would lead to a substantial reduction when we consider the absolute numbers due to the cohort size.

**Table B2** shows the prevalence rate of diabetes and hypertension by age group based on the National Population Health Survey 2022 (NPHS2022) [37] and the 5-year projected prevalence based on the No NSC scenario and the NSC scenario. Comparing the NPHS2022 data with the NSC scenario, on average, the NSC model overestimated the diabetes prevalence by 5.7% but underestimated the hypertension prevalence by 6.3%. In addition, we were interested in the difference between the NSC and no NSC scenarios; hence, any bias that appears as the difference between the data and the NSC scenario would likely also appear in the difference between the data and the no NSC scenario and thus likely cancel out when we compute the difference between the NSC and no NSC scenario.

**Table B2:** Prevalence Rate of Diabetes and Hypertension by age group.

|  | Diabetes | | | Hypertension | | | | |
| --- | --- | --- | --- | --- | --- | --- | --- | --- |
| Age (years) | Data | Model^b^ | | Data | Model^b^ | | Model + Correction^c^ | |
|  | NPHS2022^a^ | (no NSC) | (NSC) | NPHS2022^a^ | (no NSC) | (NSC) | (no NSC) | (NSC) |
| 30-39 | 1.9% | 5.9% | 5.2% | 17.4% | 8.1% | 7.5% | 9.2% | 8.5% |
| 40-49 | 5% | 11.7% | 11 % | 31.9% | 18.2% | 17.1% | 26% | 24.5% |
| 50-59 | 10.8% | 20.4% | 19.8% | 53.7% | 26.5% | 25.1% | 40.1% | 38.1% |
| 60-69 | 21.8% | 27.1% | 26.7% | 64.3% | 38.2% | 37% | 62.8% | 60.8% |
| 70-74 | 24.2% | 29.7% | 29.4% | 76.8% | 43.4% | 42.3% | 82.7% | 80.6% |

a Prevalence rate obtained from the National Population Health Survey 2022 (NPHS2022)

b Prevalence rate obtained from running a closed cohort simulation for 5 years. For example, running the model on a closed cohort aged 25 to 34 for 5 years gives a 5.2% diabetes prevalence and 7.5% hypertension prevalence in the NSC intervention scenario and a 5.9% diabetes prevalence and 8.1% hypertension prevalence.

c Recall that the NSC model models the hypertension prevalence as the population with hypertension and no diabetes. From the NHS2010 data, we had the prevalence rates of (i) the population with hypertension but no diabetes and (ii) the population with diabetes and hypertension. We multiplied column b with the ratio (ii)/(i)to obtain this column.

**(III) Model Results**

**Figure S6:** Cases averted, healthcare cost savings and quality-adjusted life-years gained when the National Steps Challenge^TM^ (NSC) is conducted yearly over 10 years, based on a mean cohort size of 654,500 participants aged 17 and above for 5 and 10 years [3].

**
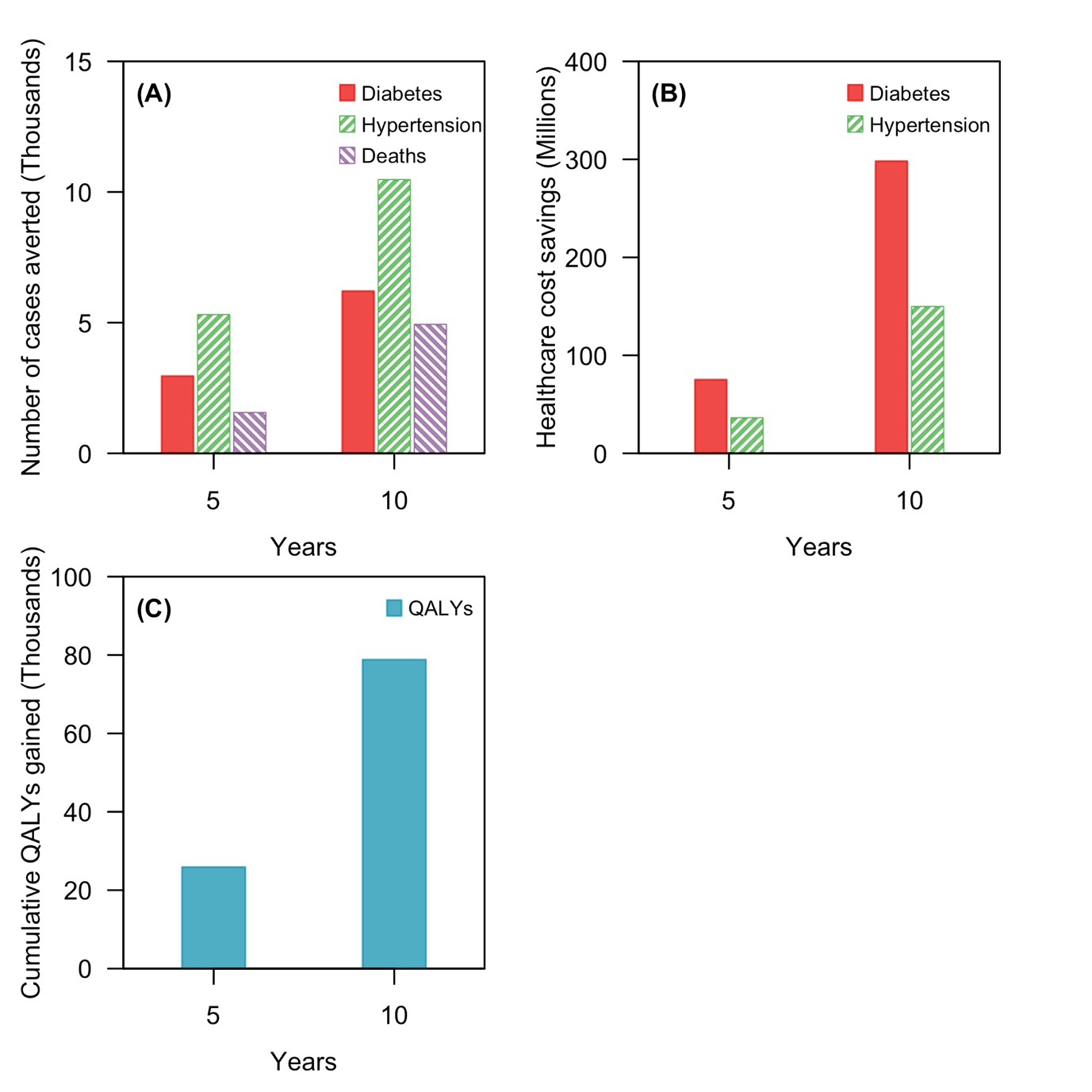
**

Panel A shows the reductions in diabetes, hypertension, and deaths. Panel B shows the reduction in healthcare costs. Panel C shows the gain in quality-adjusted life-years.

QALYs – quality-adjusted life-years.

**Figure S7:** Cumulative cases averted when the National Steps Challenge^TM^ (NSC) is conducted yearly over 10 years, based on a mean cohort size of 654,500 participants aged 17 and above [3].


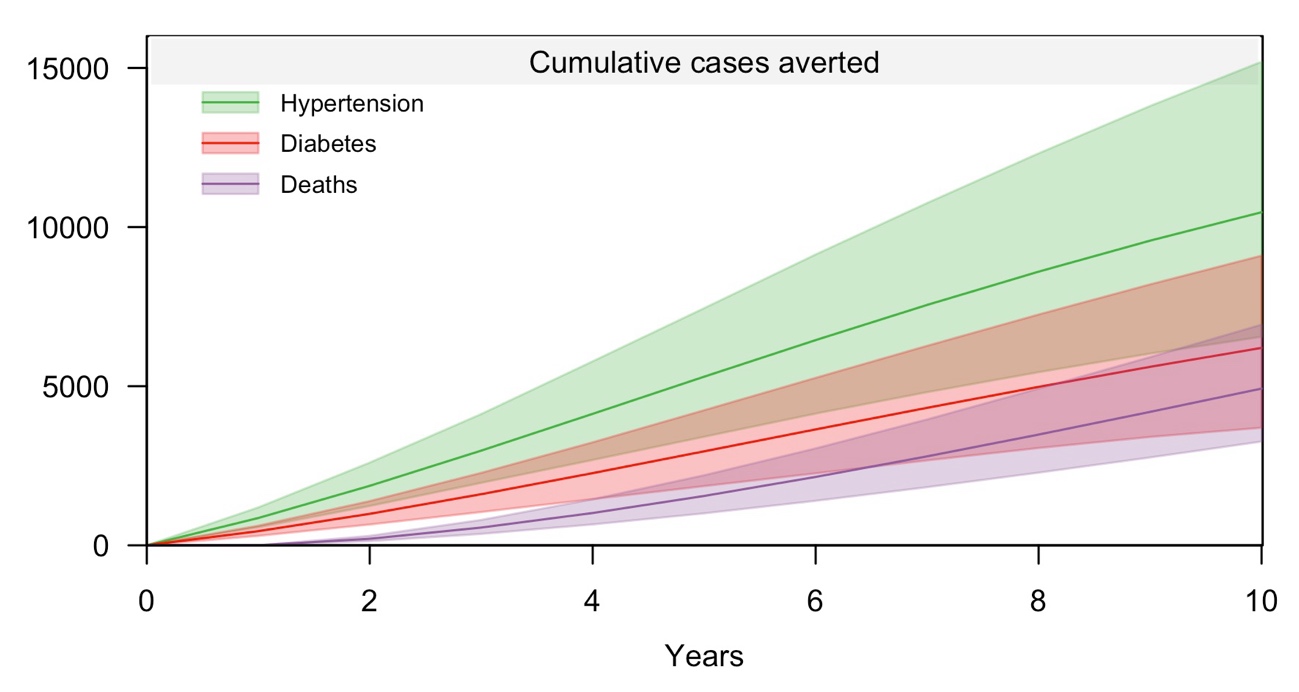


The solid lines represent the mean and the shaded regions represent the 95% credible intervals which are obtained using the 2.5 and 97.5 percentiles of the 1000 bootstrap samples.

**Figure S8:** Quality-adjusted life-years gained when the National Steps Challenge^TM^ (NSC) is conducted yearly over 10 years, based on a mean cohort size of 654,500 participants aged 17 and above [3].


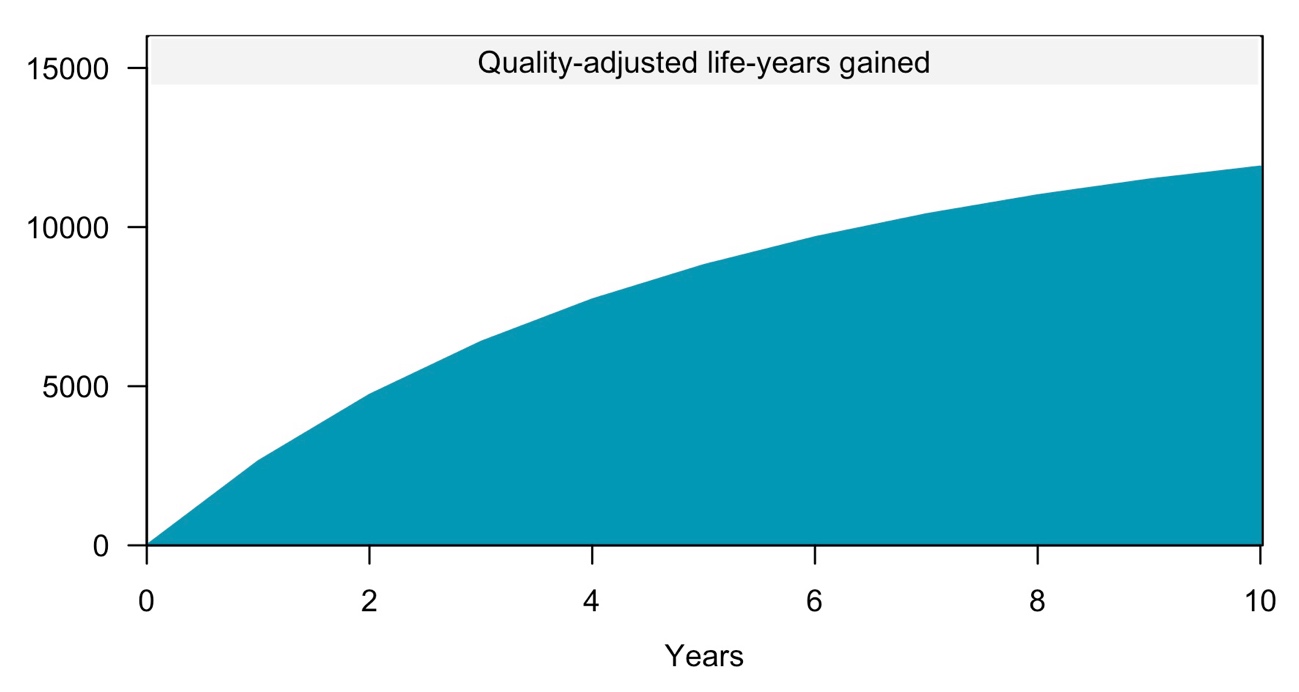


The plot shows the quality-adjusted life-years gained for each year. The area under the graph is the total quality-adjusted life-years gained from year 0.

**Figure S9:** Cumulative quality-adjusted life-years gained when the National Steps Challenge^TM^ (NSC) is conducted yearly over 10 years, based on a mean cohort size of 654,500 participants aged 17 and above [3].


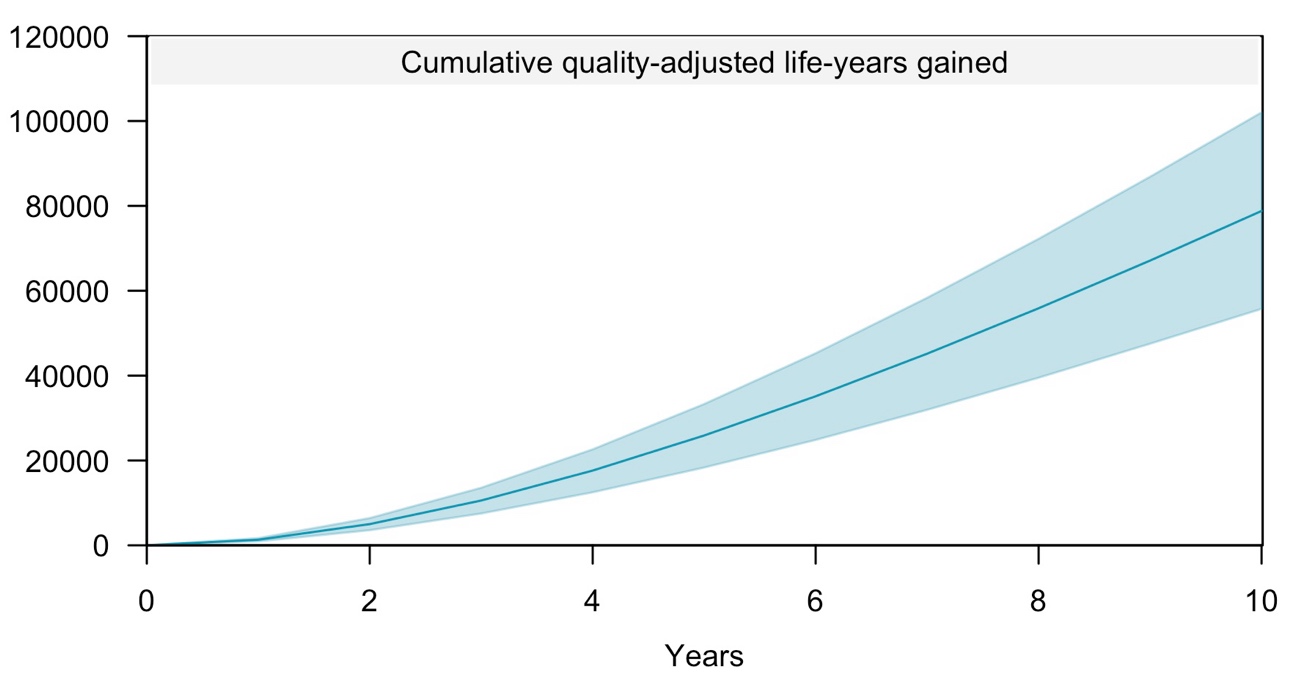


The solid lines represent the mean and the shaded regions represent the 95% credible intervals which are obtained using the 2.5 and 97.5 percentiles of the 1000 bootstrap samples.

**Figure S10:** Comparison of the cost per quality-adjusted life-year gained over time between different decay rates when the National Steps Challenge^TM^ (NSC) is conducted yearly over 10 years, based on a mean cohort size of 654,500 participants aged 17 and above.


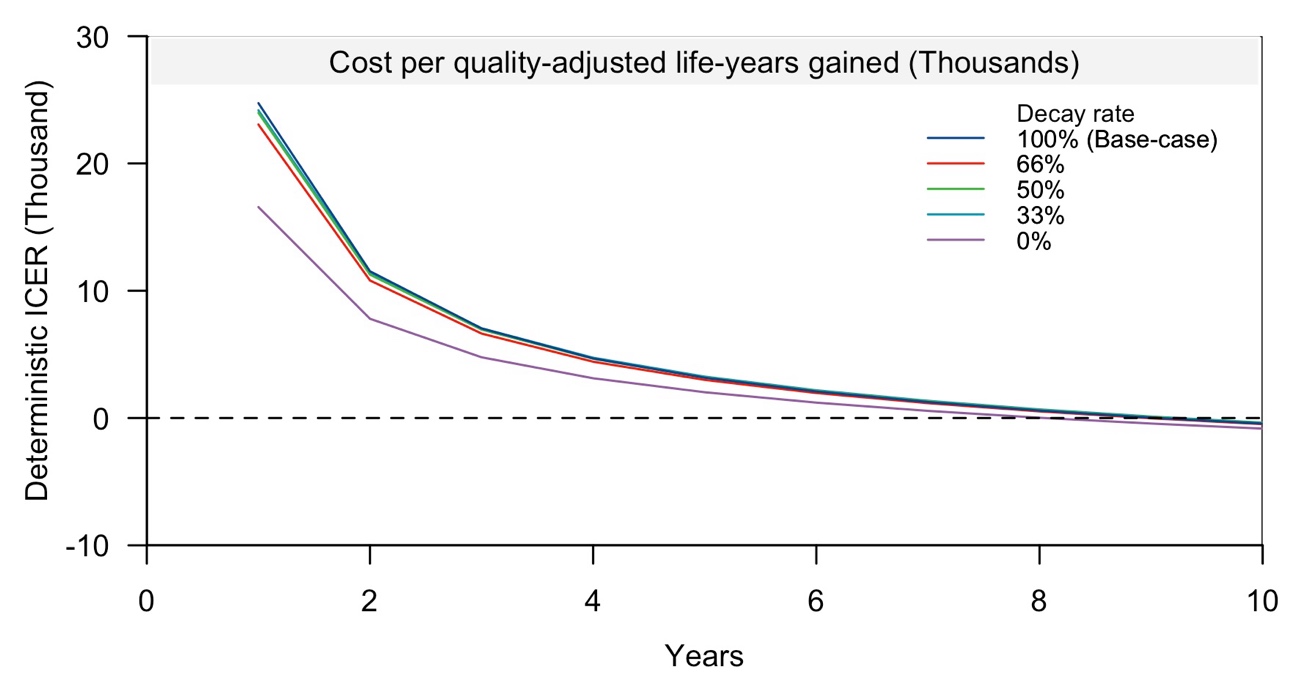


The figure shows the impact of changing the model time horizon from 1 year to 10 years has on the cost per quality-adjusted life-year gained.

The smaller the decay rate, the stronger the impact of NSC, especially in the earlier time horizon and (in general) the more cost-effective NSC is.

ICER – Incremental cost-effectiveness ratio.

**Figure S11:** Results of the probabilistic and one-way sensitivity analyses for the societal perspective when the National Steps Challenge^TM^ (NSC) is conducted yearly over 10 years, based on a mean cohort size of 654,500 participants aged 17 and above [3].

**
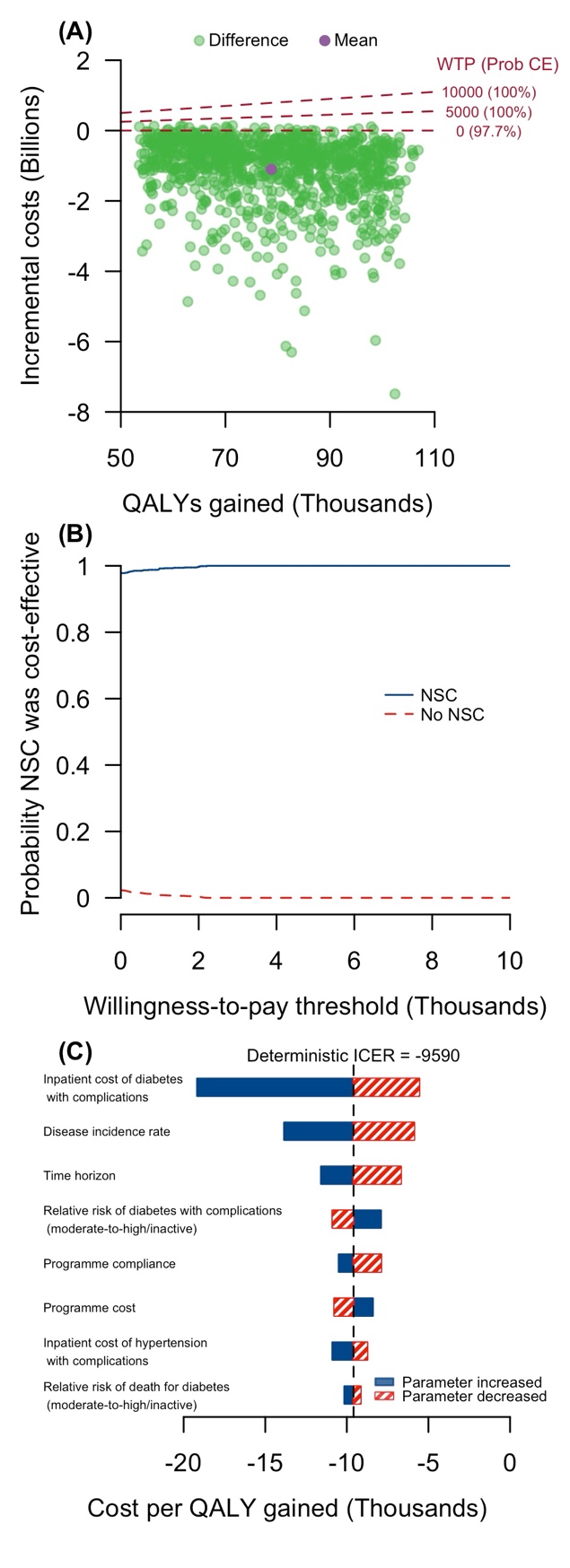
**

Panel A shows the simulated incremental costs (based on the societal perspective) and QALYs gained (green) from the probabilistic sensitivity analysis. The mean of the 1000 bootstrap samples was also plotted (purple). In each bootstrap sample, NSC is cost-effective if the simulated point (green point) is below the willingness-to-pay threshold (red dotted lines). The percentages next to the willingness to pay are the proportion of bootstrap samples below the threshold, which estimates the probability that NSC is cost-effective.

Panel B shows the cost-effectiveness acceptability curve (CEAC) based on the societal perspective. The points on the blue curve in Panel B were obtained by varying the willingness-to-pay threshold and computing the proportion of bootstrap samples in Panel A below that threshold. The red curve is obtained by subtracting the proportion of bootstrap samples below the willingness-to-pay threshold (blue curve) from one.

Panel C shows the results of the one-way sensitivity analyses (based on the societal perspective) where model parameters were varied across a range of plausible values to see the impact on the cost per QALY gained. The deterministic ICER is obtained using the model parameters' mean (or median for skewed parameters, e.g. costs). The top 8 parameters (out of 33) to which the model was most sensitive are shown. Plausible ranges were preferentially derived from reported 95% CIs or ranges, or calculated 95% CIs using standard errors as available, except for the inpatient rates, inpatient and outpatient treatment costs, programme cost, programme compliance and time horizon. The interquartile range was used for the inpatient and outpatient treatment costs, and inflating/deflating the means by 30% was used for the inpatient rates, programme cost, programme compliance and time horizon.

WTP (Prob CE) – willingness-to-pay (probability that National Steps Challenge^TM^ is cost-effective); QALY – quality-adjusted life-year; NSC – National Steps Challenge^TM^; ICER – incremental cost-effectiveness ratio.

SUPPLEMENTARY TABLES

**(I) Model Parameters**

**Table S1:** Base case parameters and ranges used in one-way and probabilistic sensitivity analyses.

| S/N | | Parameter | |  |  |  |  |  |  |  | |  |
| --- | --- | --- | --- | --- | --- | --- | --- | --- | --- | --- | --- | --- |
| 1 | | *Disease incidence rates* | |  |  |  |  |  | Follow-up (Years) | One-year mean | |  |
| 1a | | Diabetes incidence rates (Age group)^ | | Distribution^^ | Mean | S.E. | Lower* | Upper* |  |  |  | Reference |
|  | | 15 - 19 | | Beta | 0.0382 | 0.00753 | 0.0234 | 0.0530 | 6 | 0.00647 | | [25] Multi-ethnic cohort |
|  | | 20 - 24 | | Beta | 0.0440 | 0.0065 | 0.0313 | 0.0568 | 6 | 0.00748 | |  |
|  | | 25 - 29 | | Beta | 0.0499 | 0.00548 | 0.0391 | 0.0606 | 6 | 0.00849 | |  |
|  | | 30 - 34 | | Beta | 0.0557 | 0.00447 | 0.0469 | 0.0645 | 6 | 0.00951 | |  |
|  | | 35 - 39 | | Beta | 0.0615 | 0.0035 | 0.0547 | 0.0684 | 6 | 0.0105 | |  |
|  | | 40 - 44 | | Beta | 0.0691 | 0.0109 | 0.0478 | 0.0904 | 6 | 0.0119 | |  |
|  | | 45 - 49 | | Beta | 0.0703 | 0.00228 | 0.0658 | 0.0747 | 6 | 0.0121 | |  |
|  | | 50 - 54 | | Beta | 0.0795 | 0.00192 | 0.0757 | 0.0833 | 6 | 0.0137 | |  |
|  | | 55 - 59 | | Beta | 0.0858 | 0.00209 | 0.0817 | 0.0899 | 6 | 0.0148 | |  |
|  | | 60 - 64 | | Beta | 0.0905 | 0.00233 | 0.0859 | 0.0951 | 6 | 0.0157 | |  |
|  | | 65 - 69 | | Beta | 0.0882 | 0.00282 | 0.0827 | 0.0937 | 6 | 0.0153 | |  |
|  | | 70 - 74 | | Beta | 0.0880 | 0.00375 | 0.0807 | 0.0954 | 6 | 0.0152 | |  |
|  | | 75 - 79 | | Beta | 0.0837 | 0.00743 | 0.0691 | 0.0982 | 6 | 0.0145 | |  |
|  | | >79 | | Beta | 0.131 | 0.0305 | 0.0712 | 0.191 | 6 | 0.0231 | |  |
|  | |  | |  |  |  |  |  |  |  | |  |
| 1b | | Diabetes complications incidence rates (Age group)^ | | Distribution^^ | Mean | S.E. | Lower* | Upper* | Follow-up (Years) | One-year mean | | Reference |
|  | | 15 - 19 | | Beta | 0.00455** | 0.0302** | 0** | 0.0637** | 6 | 0.00076 | | [25] Multi-ethnic cohort |
|  | | 20 - 24 | | Beta | 0.00455 | 0.0302 | 0^##^ | 0.0637 | 6 | 0.00076 | |  |
|  | | 25 - 29 | | Beta | 0.0200 | 0.0255 | 0^##^ | 0.0699 | 6 | 0.00336 | |  |
|  | | 30 - 34 | | Beta | 0.0355 | 0.0208 | 0^##^ | 0.0762 | 6 | 0.00600 | |  |
|  | | 35 - 39 | | Beta | 0.0509 | 0.0162 | 0.0191 | 0.0828 | 6 | 0.00867 | |  |
|  | | 40 - 44 | | Beta | 0.0764 | 0.0871 | 0^##^ | 0.247 | 6 | 0.0132 | |  |
| 1b | | Diabetes complications incidence rates (Age group)^ | | Distribution^^ | Mean | S.E. | Lower* | Upper* | Follow-up (Years) | One-year mean | | Reference |
|  | | 45 - 49 | | Beta | 0.0697 | 0.0123 | 0.0456 | 0.0937 | 6 | 0.0120 | | [25] Multi-ethnic cohort |
|  | | 50 - 54 | | Beta | 0.0942 | 0.00983 | 0.0749 | 0.113 | 6 | 0.0164 | |  |
|  | | 55 - 59 | | Beta | 0.115 | 0.0103 | 0.0951 | 0.135 | 6 | 0.0202 | |  |
|  | | 60 - 64 | | Beta | 0.131 | 0.0115 | 0.108 | 0.153 | 6 | 0.0231 | |  |
|  | | 65 - 69 | | Beta | 0.183 | 0.0151 | 0.153 | 0.212 | 6 | 0.0331 | |  |
|  | | 70 - 74 | | Beta | 0.167 | 0.0197 | 0.129 | 0.206 | 6 | 0.0300 | |  |
|  | | 75 - 79 | | Beta | 0.259 | 0.0498 | 0.161 | 0.356 | 6 | 0.0486 | |  |
|  | | >79 | | Beta | 0.243 | 0.163 | 0^##^ | 0.563 | 6 | 0.0454 | |  |
|  | |  | |  |  |  |  |  |  |  | |  |
| 1c | | Hypertension incidence rates (Age group)^ | | Distribution^^ | Mean | S.E. | Lower* | Upper* | Follow-up (Years) | One-year mean | | Reference |
|  | | 15 - 19 | | Beta | 0.0246** | 0.0126** | 0.00** | 0.0492** | 6 | 0.00414 | | [25] Multi-ethnic cohort |
|  | | 20 - 24 | | Beta | 0.0246** | 0.0126** | 0.00** | 0.0492** | 6 | 0.00414 | |  |
|  | | 25 - 29 | | Beta | 0.0246 | 0.0126 | 0.000 | 0.0492 | 6 | 0.00414 | |  |
|  | | 30 - 34 | | Beta | 0.0581 | 0.0105 | 0.0375 | 0.0786 | 6 | 0.00992 | |  |
|  | | 35 - 39 | | Beta | 0.0916 | 0.00849 | 0.0750 | 0.108 | 6 | 0.0159 | |  |
|  | | 40 - 44 | | Beta | 0.117 | 0.0152 | 0.0875 | 0.147 | 6 | 0.0206 | |  |
|  | | 45 - 49 | | Beta | 0.162 | 0.00347 | 0.155 | 0.168 | 6 | 0.0289 | |  |
|  | | 50 - 54 | | Beta | 0.199 | 0.00329 | 0.193 | 0.206 | 6 | 0.0364 | |  |
|  | | 55 - 59 | | Beta | 0.23 | 0.00386 | 0.222 | 0.237 | 6 | 0.0426 | |  |
|  | | 60 - 64 | | Beta | 0.257 | 0.0046 | 0.248 | 0.266 | 6 | 0.0483 | |  |
|  | | 65 - 69 | | Beta | 0.288 | 0.00603 | 0.277 | 0.300 | 6 | 0.0551 | |  |
|  | | 70 - 74 | | Beta | 0.286 | 0.00809 | 0.270 | 0.302 | 6 | 0.0546 | |  |
|  | | 75 - 79 | | Beta | 0.313 | 0.0181 | 0.277 | 0.348 | 6 | 0.0606 | |  |
|  | | >79 | | Beta | 0.309 | 0.0647 | 0.182 | 0.436 | 6 | 0.0598 | |  |
|  | |  | |  |  |  |  |  |  |  | |  |
| 1d | | Hypertension complications incidence rates (Age group)^ | | Distribution^^ | Mean | S.E. | Lower* | Upper* | Follow-up (Years) | One-year mean | | Reference |
|  | | 15 - 19 | | Beta | 0.00241** | 0.00572** | 0** | 0.0136** | 6 | 0.0004 | | [25] Multi-ethnic cohort |
|  | | 20 - 24 | | Beta | 0.00241** | 0.00572** | 0** | 0.0136** | 6 | 0.0004 | |  |
|  | | 25 - 29 | | Beta | 0.00241** | 0.00572** | 0** | 0.0136** | 6 | 0.0004 | |  |
|  | | 30 - 34 | | Beta | 0.00241 | 0.00572 | 0^##^ | 0.0136 | 6 | 0.0004 | |  |
|  | | 35 - 39 | | Beta | 0.0163 | 0.00478 | 0.00689 | 0.0256 | 6 | 0.00273 | |  |
|  | | 40 - 44 | | Beta | 0.0771 | 0.0433 | 0^##^ | 0.162 | 6 | 0.0133 | |  |
|  | | 45 - 49 | | Beta | 0.0480 | 0.00499 | 0.0382 | 0.0577 | 6 | 0.00816 | |  |
|  | | 50 - 54 | | Beta | 0.0549 | 0.00356 | 0.0480 | 0.0619 | 6 | 0.00937 | |  |
|  | | 55 - 59 | | Beta | 0.0690 | 0.00373 | 0.0617 | 0.0763 | 6 | 0.0118 | |  |
|  | | 60 - 64 | | Beta | 0.0849 | 0.00415 | 0.0768 | 0.0931 | 6 | 0.0147 | |  |
|  | | 65 - 69 | | Beta | 0.100 | 0.00517 | 0.0902 | 0.110 | 6 | 0.0175 | |  |
|  | | 70 - 74 | | Beta | 0.115 | 0.00719 | 0.100 | 0.129 | 6 | 0.0201 | |  |
|  | | 75 - 79 | | Beta | 0.138 | 0.0151 | 0.109 | 0.168 | 6 | 0.0245 | |  |
|  | | >79 | | Beta | 0.134 | 0.0466 | 0.0425 | 0.225 | 6 | 0.0237 | |  |
|  | |  | |  |  |  |  |  |  |  | |  |
|  | | *Physical Activity Relative Risk for Incidence of Diseases* | | Distribution^^ | Mean | S.E. | Lower* | Upper* | - | - | | Reference |
| 2 | | Diabetes; Low PA compared to Inactive PA | | Gamma | 0.93 | - | 0.92 | 0.95 | - | - | | [14] Supplementary Cost Effectiveness |
| 3 | | Diabetes; Moderate-to-High PA compared to Inactive PA | | Gamma | 0.75 | - | 0.69 | 0.8 | - | - | |  |
| 4 | | Diabetes Complications; Low PA compared to Inactive PA | | Gamma | 0.86 | - | 0.78 | 0.93 | - | - | | [30] The effect of physical activity on mortality and cardiovascular disease in 130 000 people from 17 high-income, middle-income, and low-income countries: the PURE study. |
| 5 | | Diabetes Complications; Moderate-to-High PA compared to Inactive PA | | Gamma | 0.75 | - | 0.69 | 0.82 | - | - | |  |
| 6 | | Hypertension; Low PA compared to Inactive PA | | Gamma | 0.89 | - | 0.85 | 0.94 | - | - | | [13] Physical activity and Risk of Hypertension |
| 7 | | Hypertension; Moderate-to-High PA compared to Inactive PA | | Gamma | 0.81 | - | 0.76 | 0.85 | - | - | |  |
| 8 | | Hypertension Complications; Low PA compared to Inactive PA | | Gamma | 0.85 | - | 0.8 | 0.91 | - | - | | [33] Quantifying the Association Between Physical Activity and Cardiovascular Disease and Diabetes: A Systematic Review and Meta-Analysis. |
| 9 | | Hypertension Complications; Moderate-to-High PA compared to Inactive PA | | Gamma | 0.81 | - | 0.74 | 0.88 | - | - | |  |
|  | | *Physical Activity Relative Risk for Mortality from Diseases* | | Distribution^^ | Mean | S.E. | Lower* | Upper* | - | - | | Reference |
| 10 | | Healthy; Low PA compared to Inactive PA | | Gamma | 0.8 | - | 0.74 | 0.87 | - | - | | [30] The effect of physical activity on mortality and cardiovascular disease in 130 000 people from 17 high-income, middle-income, and low-income countries: the PURE study. |
| 11 | | Healthy; Moderate-to-High PA compared to Inactive PA | | Gamma | 0.65 | - | 0.6 | 0.71 | - | - | |  |
| 12 | | Diabetes; Low PA compared to Inactive PA | | - | 0.8 | - | 0.67 | 0.95 | - | - | |  |
| 13 | | Diabetes; Moderate-to-High PA compared to Inactive PA | | Gamma | 0.68 | - | 0.57 | 0.82 | - | - | |  |
| 14 | | Hypertension; Low PA compared to Inactive PA | | Gamma | 0.85 | - | 0.76 | 0.95 | - | - | |  |
| 15 | | Hypertension; Moderate-to-High PA compared to Inactive PA | | Gamma | 0.68 | - | 0.61 | 0.76 | - | - | |  |
|  | |  | |  |  |  |  |  |  |  | |  |
|  | | *Relative Risk of Mortality for Diseases* | | Distribution^^ | Mean | S.E. | Lower* | Upper* | - | - | | Reference |
| 16 | | Diabetes compared to Healthy | | Gamma | 1.89 | - | 1.74 | 2.04 | - | - | | [31] Association of Diabetes With All-Cause and Cause-Specific Mortality in Asia: A Pooled Analysis of More Than 1 Million Participants |
| 17 | | Diabetes Complications compared to Healthy | | Gamma | 2.57 | - | 2.19 | 3.02 | - | - | |  |
| 18 | | Hypertension compared to Healthy | | Gamma | 1.4 | - | 1 | 1.8 | - | - | | [32] Hypertension, concurrent cardiovascular risk factors and mortality: the Singapore Cardiovascular Cohort Study |
| 19 | | Hypertension Complications compared to Healthy | | Gamma | 2.3 | - | 1.9 | 3 | - | - | |  |
|  | |  | |  |  |  |  |  |  |  | |  |
|  | | *Ratio of total costs (direct and indirect costs) to direct costs* | | - | Value | - | - | - | - | - | | Reference |
|  | | Diabetes | | - | 3.77 | - | - | - | - | - | | [22] Current and future economic burden of diabetes among working-age adults in Asia: conservative estimates for Singapore from 2010-2050. |
|  | | Hypertension | | - | 1.94 | - | - | - | - | - | | [23] A global perspective on the costs of hypertension: a systematic review. |
|  | *Inpatient costs based on unsubsidised rates* | | Distribution^^ | | Median | S.E. | Lower^#^ | Upper^#^ | - | - | Reference | |
| 20 | Diabetes | | Gamma | | 6770 | - | 4300 | 10600 | - | - | [19] Ministry of Health Fee Benchmark, https://www.moh.gov.sg/cost-financing/fee-benchmarks-and-bill-amount-information/ | |
| 21 | Diabetes with complications | | Gamma | | 13400 | - | 7760 | 26700 | - | - |  |  |
| 22 | Hypertension | | Gamma | | 4760 | - | 3160 | 7760 | - | - |  |  |
| 23 | Hypertension with complications | | Gamma | | 9280 | - | 6070 | 14200 | - | - |  |  |
|  |  | |  | |  |  |  |  |  |  |  | |
|  | *Outpatient costs* | | Distribution^^ | | Mean | S.E. | Lower^#^ | Upper^#^ | - | - | Reference | |
| 24 | Diabetes | | Gamma | | 691 | 481 | 337 | 928 | - | - | [20] Direct Medical Cost of Type 2 Diabetes in Singapore | |
| 25 | Hypertension | | Gamma | | 456 | 222 | 294 | 580 | - | - | [21] Healthcare cost of patients with multiple chronic diseases in Singapore public primary care setting | |
|  |  | |  | |  |  |  |  |  |  |  | |
|  | *Proportion of inpatient cases* | | Distribution^^ | | Mean | S.E. | Lower | Upper | - | - | Reference | |
| 26 | Diabetes | | Uniform | | 0.166 | - | 0.116 | 0.216 | - | - | [20] Direct Medical Cost of Type 2 Diabetes in Singapore | |
|  | Diabetes with complications | | - | | 1 | - | - | - | - | - | - | |
| 27 | Hypertension | | Uniform | | 0.125 | - | 0.0875 | 0.163 | - | - | [10] National Population Health Survey 2020 | |
|  | Hypertension with complications | | - | | 1 | - | - | - | - | - | - | |
|  |  | |  | |  |  |  |  |  |  |  | |
|  | *Utilities* | | Distribution^^ | | Mean | S.E. | Lower | Upper | - | - | Reference | |
|  | Healthy and Inactive PA | | - | | 0.951 | - | - | - | - | - | See section 3.1.  **Inactive and Moderate-to-High PA**  [14] Cost-effectiveness of physical activity interventions in adolescents: model development and illustration using two exemplar interventions;  [15] Population norms for the EQ-5D index scores using Singapore preference weights  **Low PA**  [15] Population norms for the EQ-5D index scores using Singapore preference weights | |
|  | Healthy and Low PA | | - | | 0.973 | - | - | - | - | - |  |  |
|  | Healthy and Moderate-to-High PA | | - | | 0.993 | - | - | - | - | - |  |  |
| 28a | Diabetes and Inactive PA | | - | | 0.850 | - | - | - | - | - |  |  |
| 28b | Diabetes and Low PA | | Beta | | 0.87 | 0.02 | 0.828 | 0.907 | - | - |  |  |
| 28c | Diabetes and Moderate-to-High PA | | - | | 0.888 | - | - | - | - | - |  |  |
| 29a | Hypertension and Inactive PA | | - | | 0.889 | - | - | - | - | - |  |  |
| 29b | Hypertension and Low PA | | Beta | | 0.91 | 0.01 | 0.889 | 0.929 | - | - |  |  |
| 29c | Hypertension and Moderate-to-High PA | | - | | 0.929 | - | - | - | - | - |  |  |
|  | Diabetes Complications | | - | | 0.747 | - | - | - | - | - | See Section 3.1 | |
| 30 | Hypertension Complications | | Beta | | 0.65 | 0.09 | 0.465 | 0.814 | - | - | [15] Population norms for the EQ-5D index scores using Singapore preference weights | |
|  |  | |  | |  |  |  |  |  |  |  | |
|  |  | | Distribution^^ | | Mean | S.E. | Lower | Upper | - | - | Reference | |
| 31 | Programme cost (Millions) | | Uniform | | 36.0 | - | 25.2 | 46.8 | - | - | See Section 3.2. | |
| 32 | Programme compliance | | Uniform | | 0.3 | - | 0.21 | 0.39 | - | - | See Section 1. | |
| 33 | Time Horizon | | - | | 10 | - | 7 | 13 | - | - | - | |

S.E.: Standard error; ^ Disease incidence rates were adjusted for gender, race, education attainment, 5-year age group, BMI, marital status and smoking; ^^ For probability sensitivity analyses; *Based on 95% confidence interval; ** As incidence rates was projected to be negative, we used the youngest age group with a positive incidence rate as the estimate;^#^ based on Interquartile range; PA: physical activity; ^##^ Whenever the lower bound of the confidence interval were estimated to be negative, they were replaced with 0.

**Table S2:** Age distribution for Singapore Resident Population 2020; obtained from Singapore Department of Statistics [7].

| Total Residents | 4,040,000 |  |  |  |
| --- | --- | --- | --- | --- |
| Age group | Number of residents, in thousands |  | Age group | Number of residents, in thousands |
| 0 - 4 Years | 183 |  | 50 - 54 Years | 296 |
| 5 - 9 Years | 199 |  | 55 - 59 Years | 306 |
| 10 - 14 Years | 206 |  | 60 - 64 Years | 285 |
| 15 - 19 Years | 215 |  | 65 - 69 Years | 229 |
| 20 - 24 Years | 245 |  | 70 - 74 Years | 170 |
| 25 - 29 Years | 287 |  | 75 - 79 Years | 91.0 |
| 30 - 34 Years | 298 |  | 80 - 84 Years | 66.5 |
| 35 - 39 Years | 300 |  | 85 - 89 Years | 36.6 |
| 40 - 44 Years | 299 |  | 90 Years & Over | 20.9 |
| 45 - 49 Years | 312 |  |

**Table S3:** Life table for Singapore Resident Population 2018; obtained from Singapore Department of Statistics [26].

| Age | Death Probability |  |  |  |  |  |  |  |  |  |
| --- | --- | --- | --- | --- | --- | --- | --- | --- | --- | --- |
| 17 | 0.00018 |  |  |  |  |  |  |  |  |  |
| 18 | 0.00020 |  | Age | Death Probability |  | Age | Death Probability |  | Age | Death Probability |
| 19 | 0.00021 |  |  |  |
| 20 | 0.00022 |  | 40 | 0.00064 |  | 60 | 0.00519 |  | 80 | 0.04141 |
| 21 | 0.00024 |  | 41 | 0.00070 |  | 61 | 0.00568 |  | 81 | 0.04609 |
| 22 | 0.00025 |  | 42 | 0.00078 |  | 62 | 0.00621 |  | 82 | 0.05118 |
| 23 | 0.00025 |  | 43 | 0.00088 |  | 63 | 0.00680 |  | 83 | 0.05712 |
| 24 | 0.00025 |  | 44 | 0.00100 |  | 64 | 0.00743 |  | 84 | 0.06388 |
| 25 | 0.00025 |  | 45 | 0.00112 |  | 65 | 0.00806 |  | 85 | 0.07116 |
| 26 | 0.00025 |  | 46 | 0.00124 |  | 66 | 0.00875 |  | 86 | 0.07914 |
| 27 | 0.00025 |  | 47 | 0.00138 |  | 67 | 0.00960 |  | 87 | 0.08789 |
| 28 | 0.00027 |  | 48 | 0.00155 |  | 68 | 0.01072 |  | 88 | 0.09746 |
| 29 | 0.00029 |  | 49 | 0.00174 |  | 69 | 0.01198 |  | 89 | 0.10790 |
| 30 | 0.00031 |  | 50 | 0.00193 |  | 70 | 0.01328 |  | 90 | 0.11928 |
| 31 | 0.00034 |  | 51 | 0.00213 |  | 71 | 0.01466 |  | 91 | 0.13163 |
| 32 | 0.00036 |  | 52 | 0.00235 |  | 72 | 0.01630 |  | 92 | 0.14502 |
| 33 | 0.00038 |  | 53 | 0.00261 |  | 73 | 0.01834 |  | 93 | 0.15951 |
| 34 | 0.00040 |  | 54 | 0.00290 |  | 74 | 0.02057 |  | 94 | 0.17513 |
| 35 | 0.00042 |  | 55 | 0.00319 |  | 75 | 0.02285 |  | 95 | 0.19193 |
| 36 | 0.00044 |  | 56 | 0.00350 |  | 76 | 0.02530 |  | 96 | 0.20996 |
| 37 | 0.00047 |  | 57 | 0.00385 |  | 77 | 0.02833 |  | 97 | 0.22925 |
| 38 | 0.00052 |  | 58 | 0.00426 |  | 78 | 0.03229 |  | 98 | 0.24983 |
| 39 | 0.00058 |  | 59 | 0.00472 |  | 79 | 0.03679 |  | 99 | 0.27173 |

**(II) Model Results**

**Table S4:** Costs saved and quality-adjusted life-years gained based on probabilistic sensitivity analysis and deterministic analysis when the National Steps Challenge^TM^ (NSC) is conducted yearly over 10 years, with different initial physical activity prevalence rates, based on a mean cohort size of 654,500 participants aged 17 and above.

| Analysis | Probabilistic | Deterministic | Deterministic | Deterministic | Deterministic |
| --- | --- | --- | --- | --- | --- |
| Initial physical activity level (%) | Base Case | | Low PA scenario | High PA scenario | 2013 scenario |
| Inactive | 39.1 | 39.1 | 50 | 20 | 51.4 |
| Low | 40.5 | 40.5 | 25 | 40 | 38 |
| Moderate-to-High | 20.4 | 20.4 | 25 | 40 | 10.6 |
|  |  |  |  |  |  |
| Reduction in cases |  |  |  |  |  |
| Diabetes | 6,200  (3,700 to 9,100) | 5,970 | 5,780 | 6,000 | 5,920 |
| Hypertension | 10,500  (6,550 to 15,200) | 10,100 | 10,300 | 9,870 | 10,200 |
| Death | 4,930  (3,260 to 6,930) | 5,010 | 5,370 | 3,290 | 6,010 |
|  |  |  |  |  |  |
| Cost Savings,  in millions of SGD |  |  |  |  |  |
| Diabetes | 298  (34.7 to 925) | 216 | 216 | 215 | 216 |
| Hypertension | 150  (46.5 to 328) | 131 | 133 | 130 | 131 |
| Total | 448  (132 to 1090) | 346 | 348 | 346 | 347 |
|  |  |  |  |  |  |
| QALY gained | 78,800  (55,700 to 102,000) | 78,700 | 84,600 | 51,400 | 94,500 |
| Cost per QALY gained, in SGD | -1,660  (-9,870 to 2,860) | -442 | -432 | -661 | -376 |

Deterministic analysis was conducted by using the mean (or median for skewed parameters, e.g. costs) of the model parameters. We adopted the health system perspective. A 3% discount rate was assumed. It was also assumed that it costs SGD36 million to conduct NSC per year. For the 2013 scenario, we considered a time trend in the physical activity prevalence rates. More details can be found in Section 9.3.

PA – Physical activity; SGD – Singapore dollar; QALY – quality-adjusted life-year; NSC – National Steps Challenge^TM^.

**Table S5:** Costs saved due to differential costs from different physical activity levels due to better management of diseases using deterministic analysis when the National Steps Challenge^TM^ (NSC) is conducted yearly over 10 years, based on a mean cohort size of 654,500 participants aged 17 and above.

| Adjustment of costs due to physical activity | **0%** | **2.5%** | **5%** | **10%** |
| --- | --- | --- | --- | --- |
| Cost Savings,  in millions of SGD |  |  |  |  |
| Diabetes | 216 | 233 | 250 | 284 |
| Hypertension | 131 | 145 | 159 | 187 |
| Total | 346 | 378 | 409 | 471 |
|  |  |  |  |  |
| QALY gained | 78,700 | | | |
| Cost per QALY gained, in SGD | -442 | -837 | -1,230 | -2,020 |

Deterministic analysis was conducted by using the mean (or median for skewed parameters, e.g. costs) of the model parameters. We adopted the health system perspective. A 3% discount rate was assumed. It is also assumed that it costs SGD36 million to conduct NSC per year.

SGD – Singapore dollar; QALY – quality-adjusted life-year; NSC – National Steps Challenge^TM^.

**Table S6:** Costs saved and quality-adjusted life-years gained due to different discount rates using deterministic analysis when the National Steps Challenge^TM^ (NSC) is conducted yearly over 10 years, based on a mean cohort size of 654,500 participants aged 17 and above.

| Discount rate | **0%** | **3%** | **5%** | **10%** |
| --- | --- | --- | --- | --- |
| Cost Savings,  in millions of SGD |  |  |  |  |
| Diabetes | 263 | 216 | 190 | 141 |
| Hypertension | 160 | 131 | 115 | 84.9 |
| Total | 423 | 346 | 305 | 226 |
|  |  |  |  |  |
| QALY gained | 94,800 | 78,700 | 69,900 | 53,100 |
| Cost per QALY gained, in SGD | -668 | -442 | -285 | 123 |

Deterministic analysis was conducted by using the mean (or median for skewed parameters, e.g. costs) of the model parameters. We adopted the health system perspective. It was assumed that it costs SGD36 million to conduct NSC per year.

SGD – Singapore dollar; QALY – quality-adjusted life-year; NSC – National Steps Challenge^TM^.

SUPPLEMENTARY REFERENCES

1. As a logarithmic transformation of the response variable did not improve the linearity assumption of the regression model, no transformation was done. [↑](#footnote-ref-2)
2. From Figure S5, in the no-intervention situation (no NSC), participants transition into the “complications” state, hence the physical activity prevalence decreases. Furthermore, participants in the “Inactive” physical activity level have a higher probability to transition to the “complications” state compared to participants in the “Low” physical activity level, whom themselves have a higher probability to transition to the “complications” state compared to participants in the “Moderate-to-High” physical activity level. [↑](#footnote-ref-3)
