# Supplementary material for: A Nationwide Physical Activity Intervention for 654,500 Adults in Singapore: Cost-Utility Analysis
Source: JMIR Public Health Surveill. 2024 Oct 4;10:e46178. doi: 10.2196/46178 (PMC11468974; doi:10.2196/46178)

## Slide 1
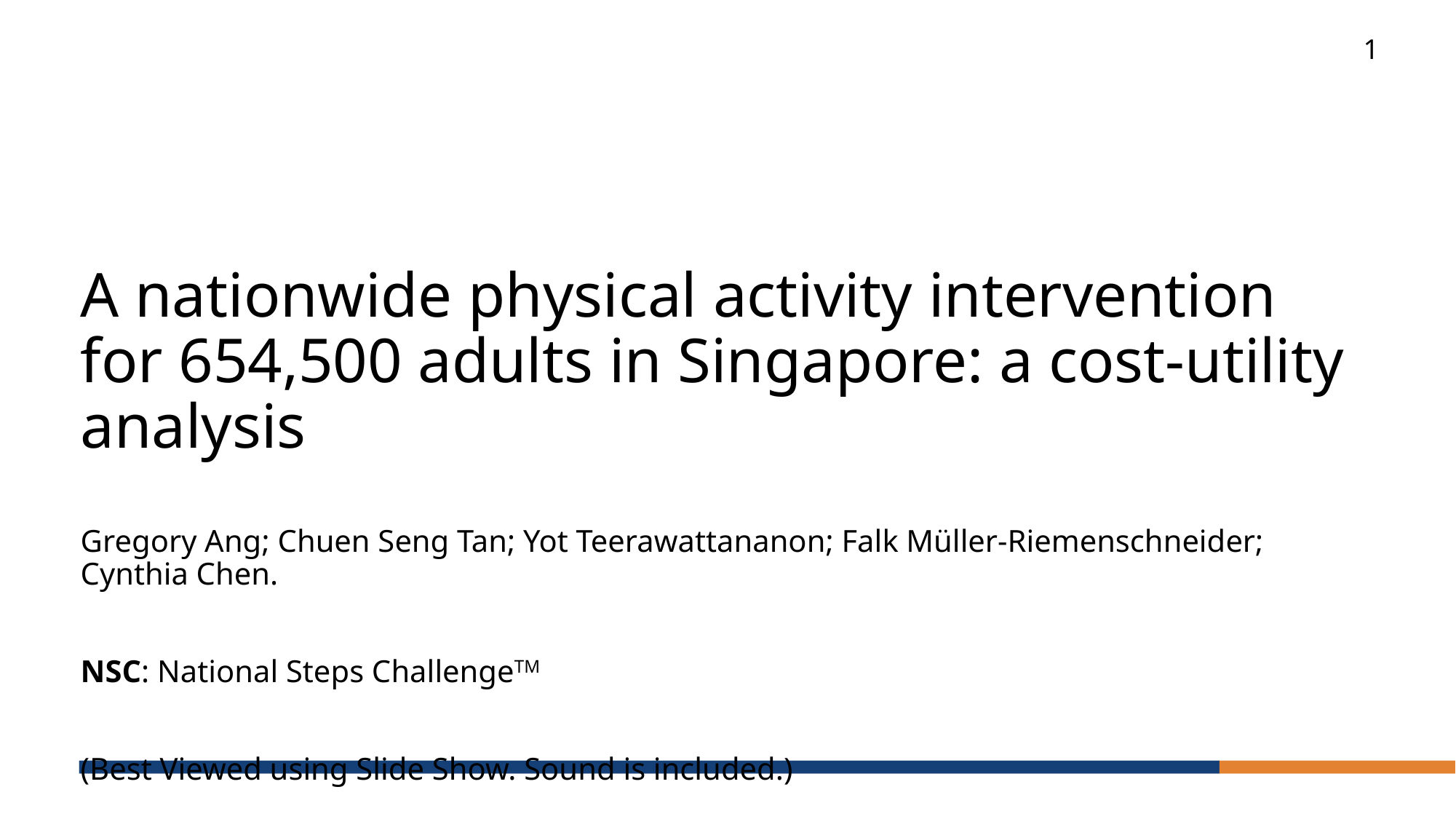

A nationwide physical activity intervention for 654,500 adults in Singapore: a cost-utility analysis
Gregory Ang; Chuen Seng Tan; Yot Teerawattananon; Falk Müller-Riemenschneider; Cynthia Chen.
NSC: National Steps ChallengeTM
(Best Viewed using Slide Show. Sound is included.)

## Slide 2
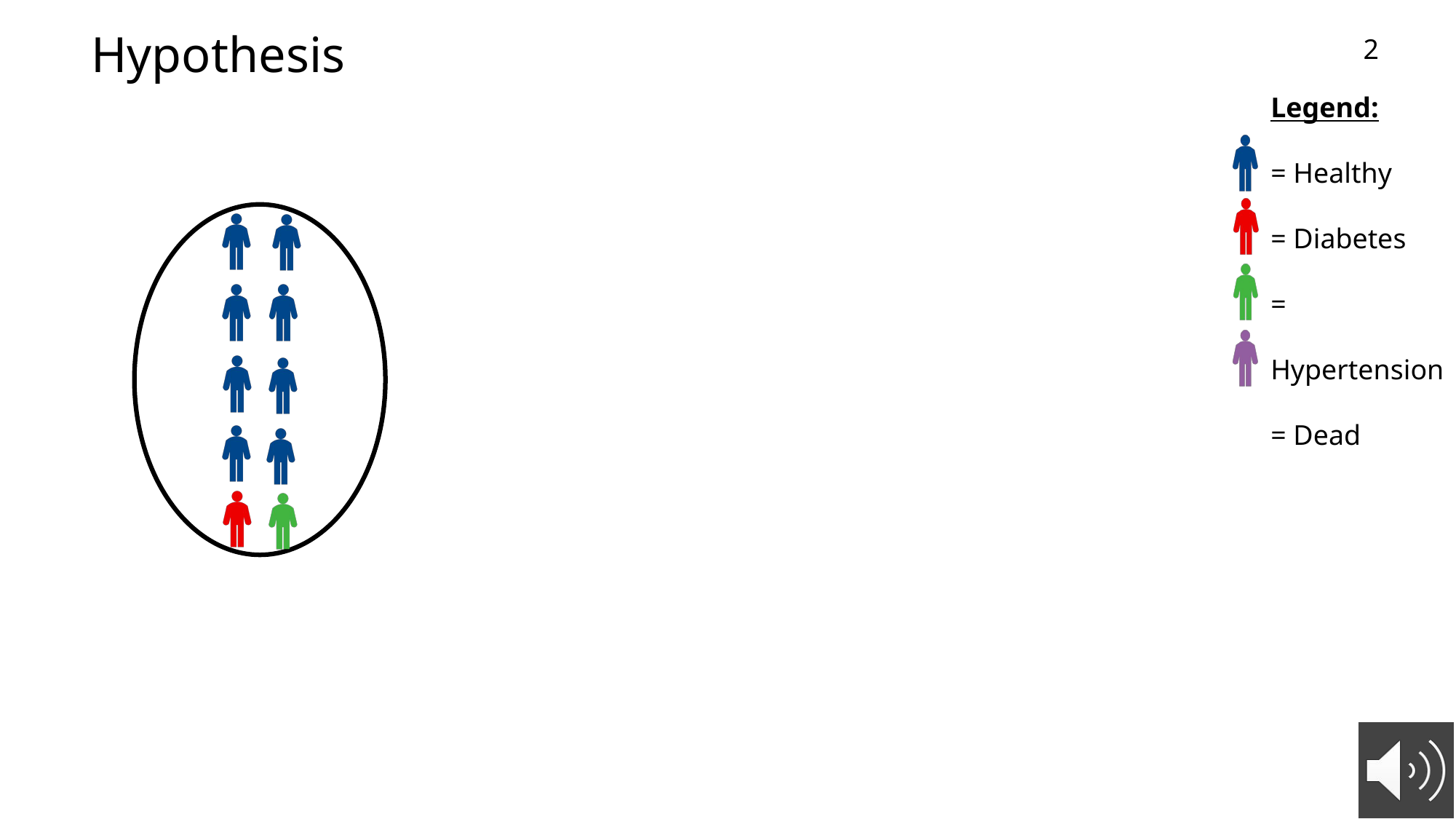

# Hypothesis
Legend:
= Healthy
= Diabetes
= Hypertension
= Dead

## Slide 3
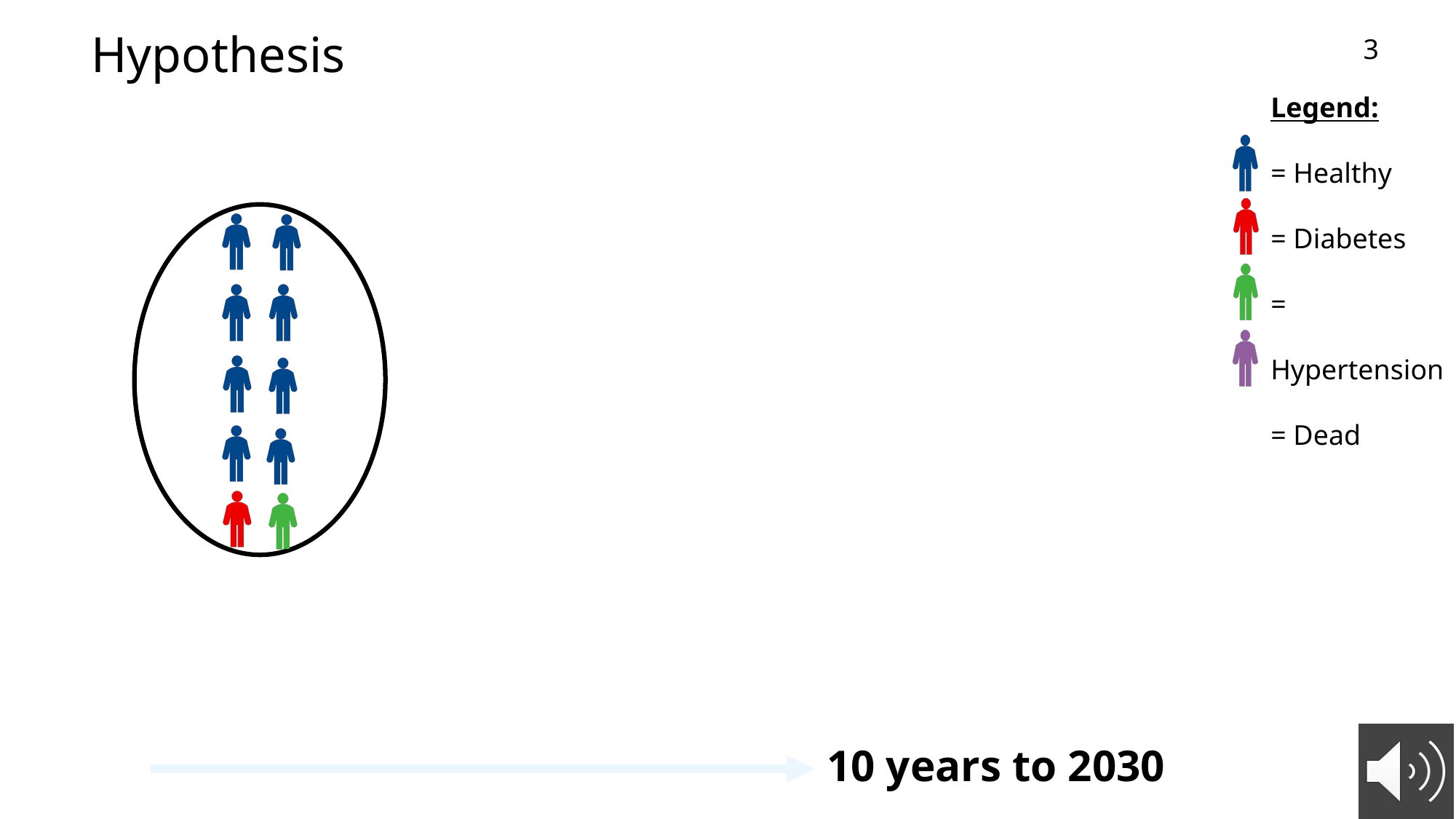

# Hypothesis
Legend:
= Healthy
= Diabetes
= Hypertension
= Dead
10 years to 2030

## Slide 4
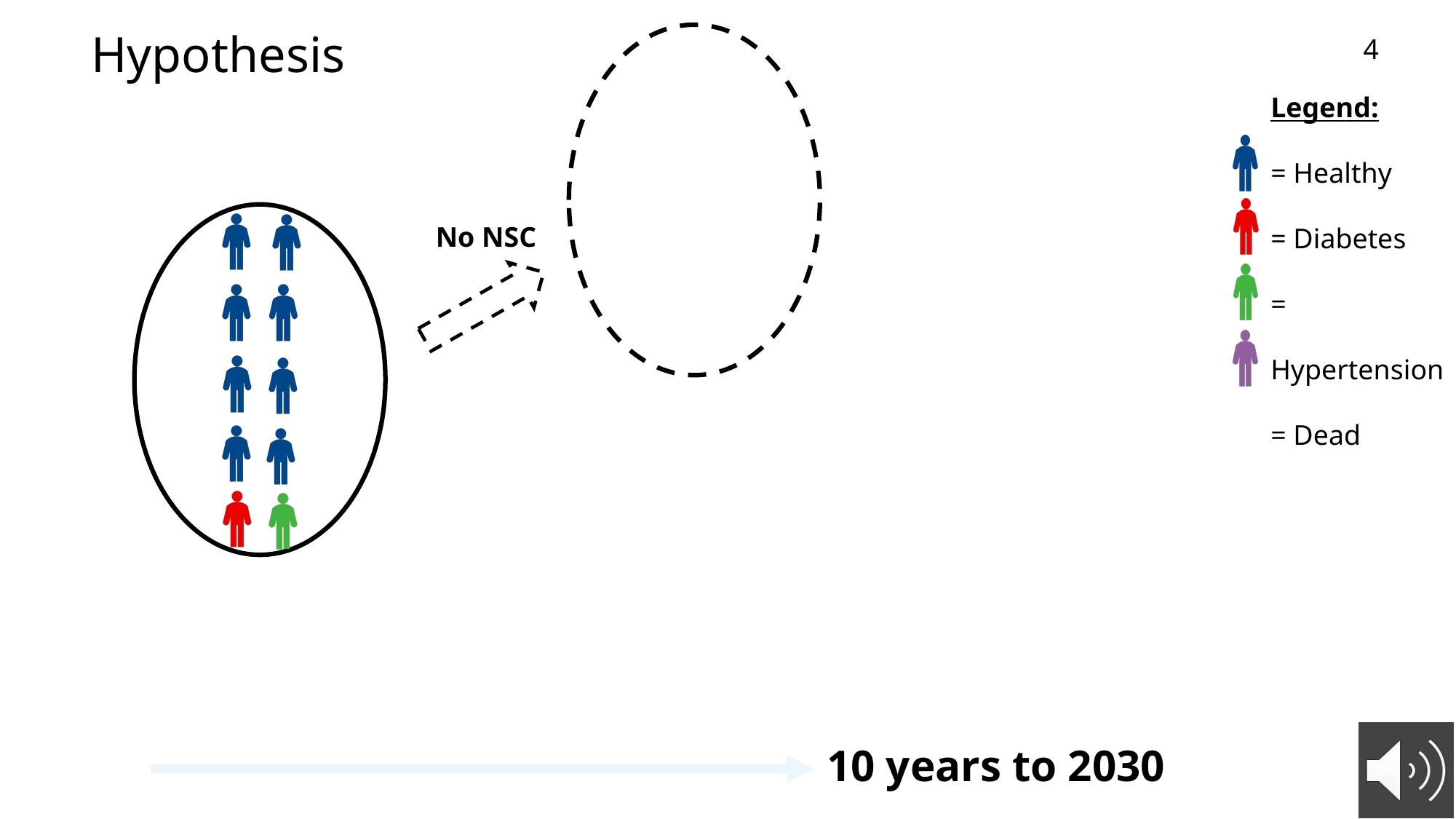

# Hypothesis
Legend:
= Healthy
= Diabetes
= Hypertension
= Dead
No NSC
10 years to 2030

## Slide 5
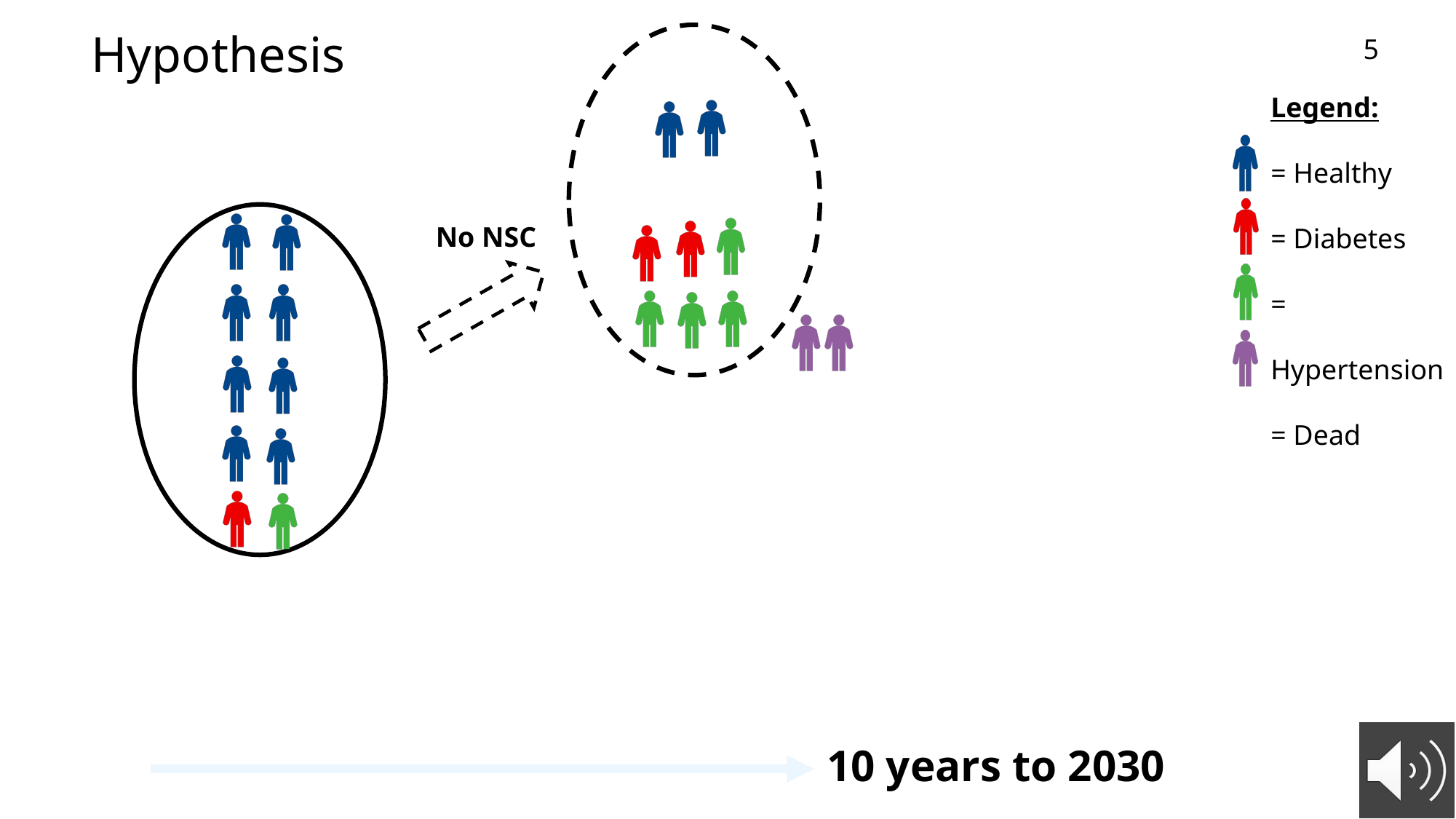

# Hypothesis
Legend:
= Healthy
= Diabetes
= Hypertension
= Dead
No NSC
10 years to 2030

## Slide 6
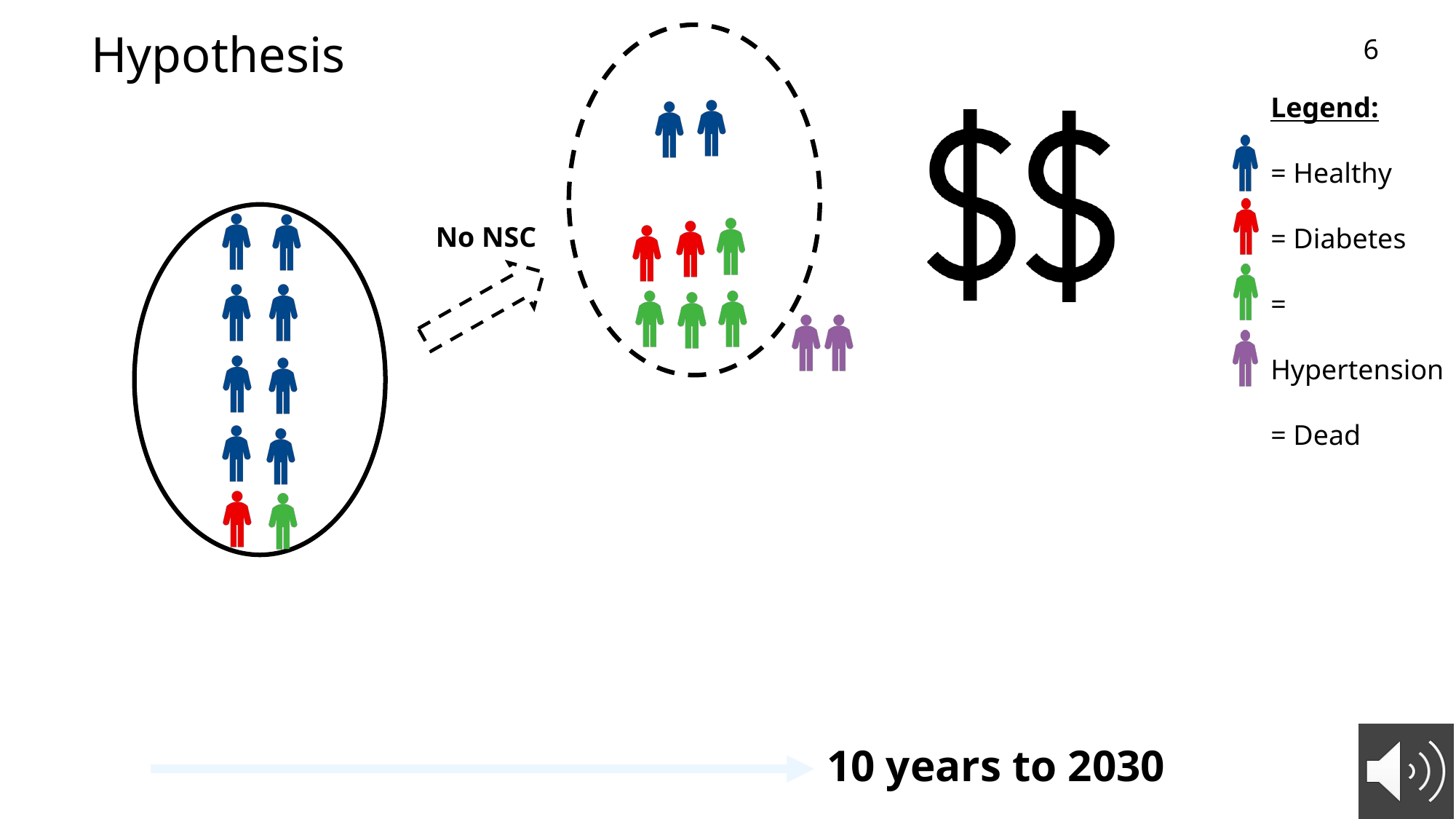

# Hypothesis
Legend:
= Healthy
= Diabetes
= Hypertension
= Dead
No NSC
10 years to 2030

## Slide 7
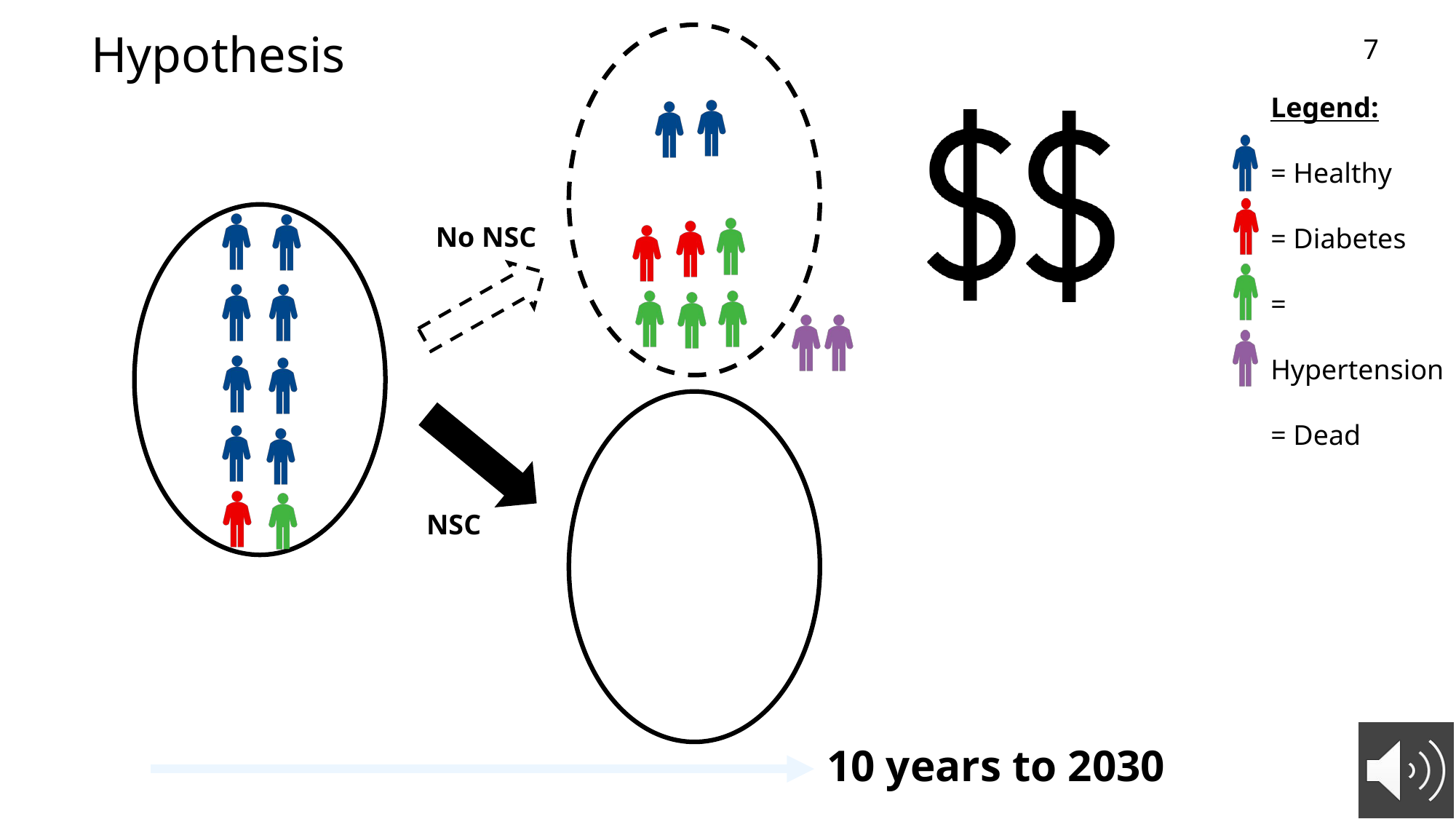

# Hypothesis
Legend:
= Healthy
= Diabetes
= Hypertension
= Dead
No NSC
NSC
10 years to 2030

## Slide 8
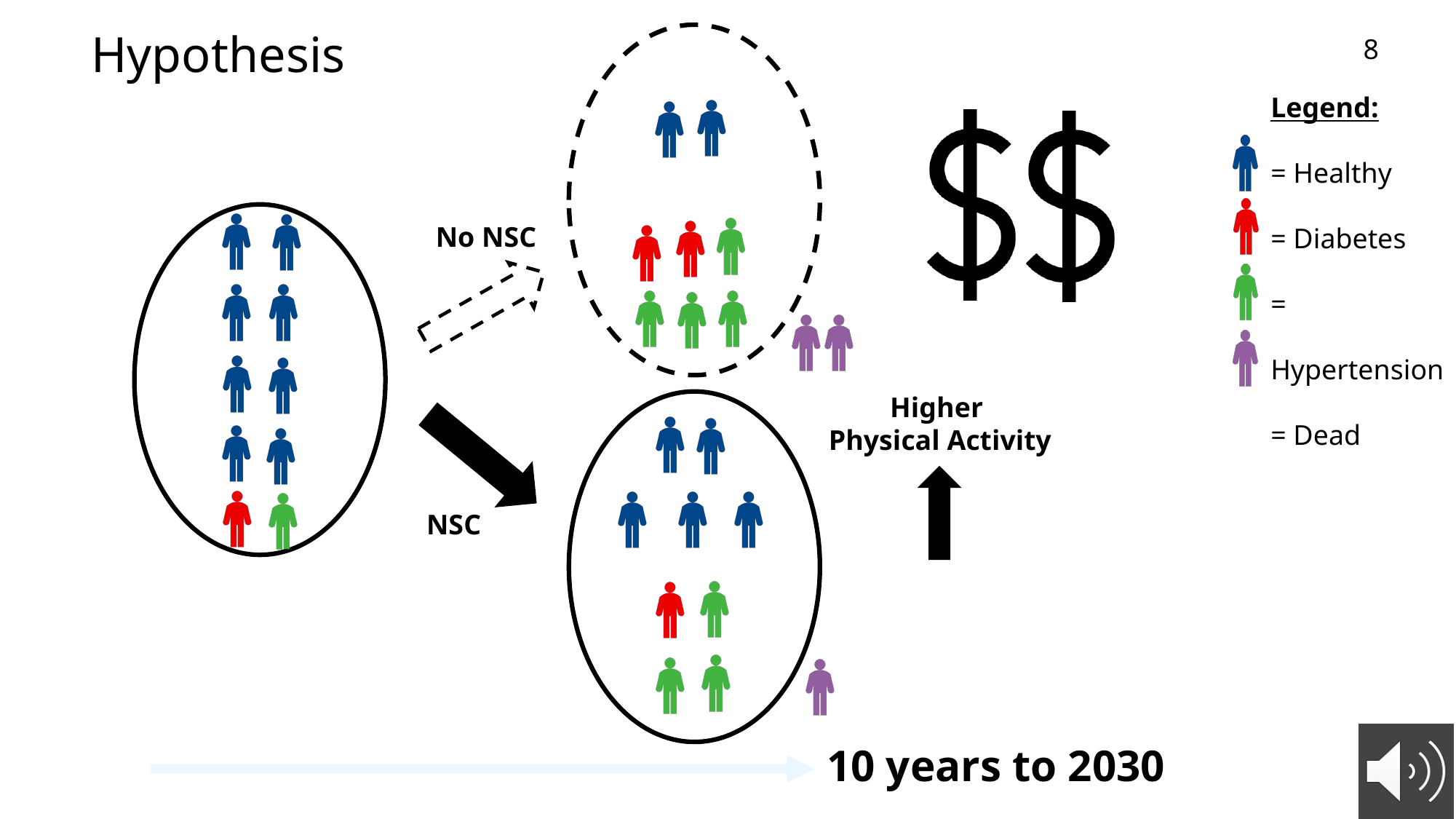

# Hypothesis
Legend:
= Healthy
= Diabetes
= Hypertension
= Dead
No NSC
Higher
Physical Activity
NSC
10 years to 2030

## Slide 9
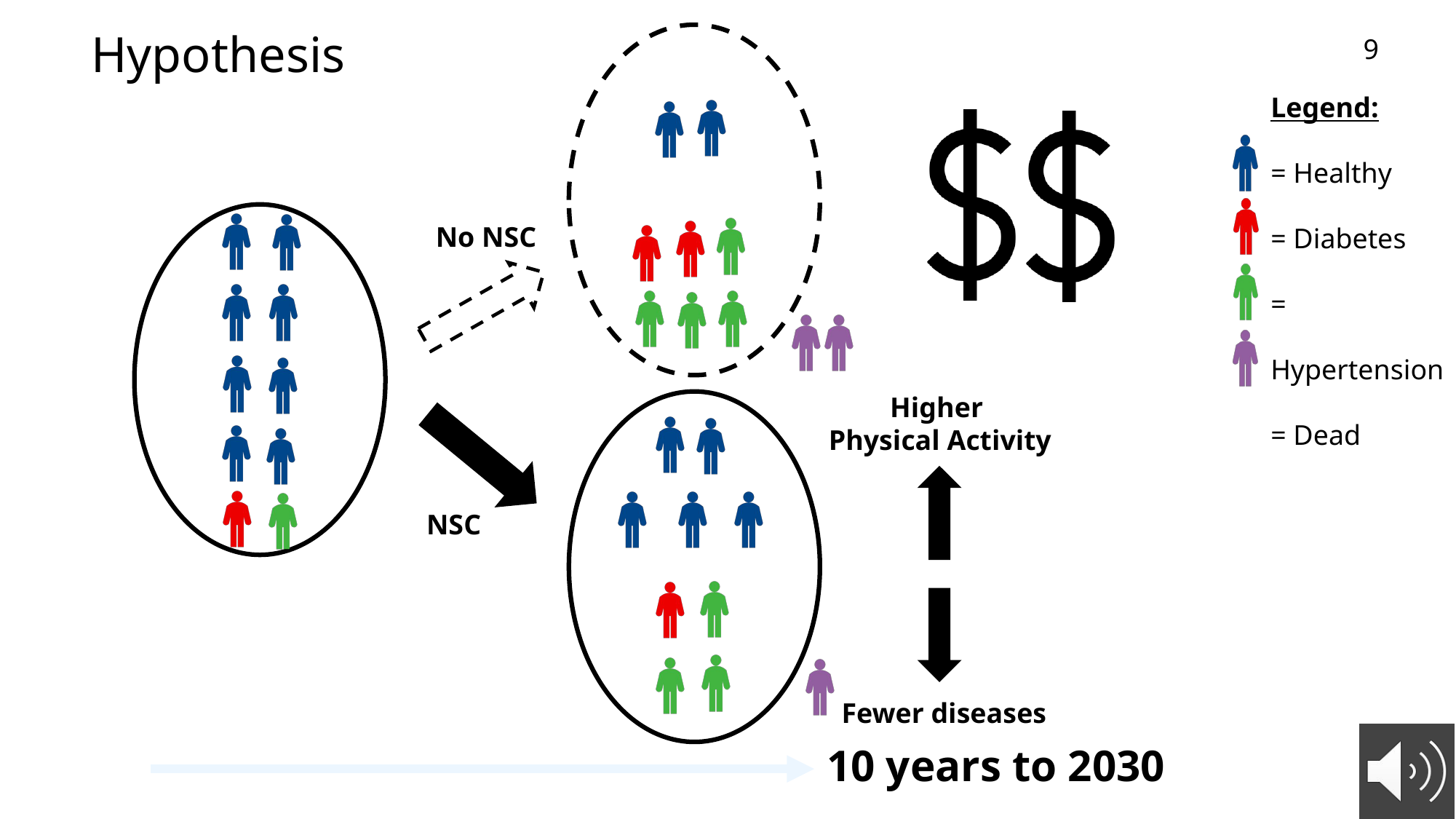

# Hypothesis
Legend:
= Healthy
= Diabetes
= Hypertension
= Dead
No NSC
Higher
Physical Activity
NSC
Fewer diseases
10 years to 2030

## Slide 10
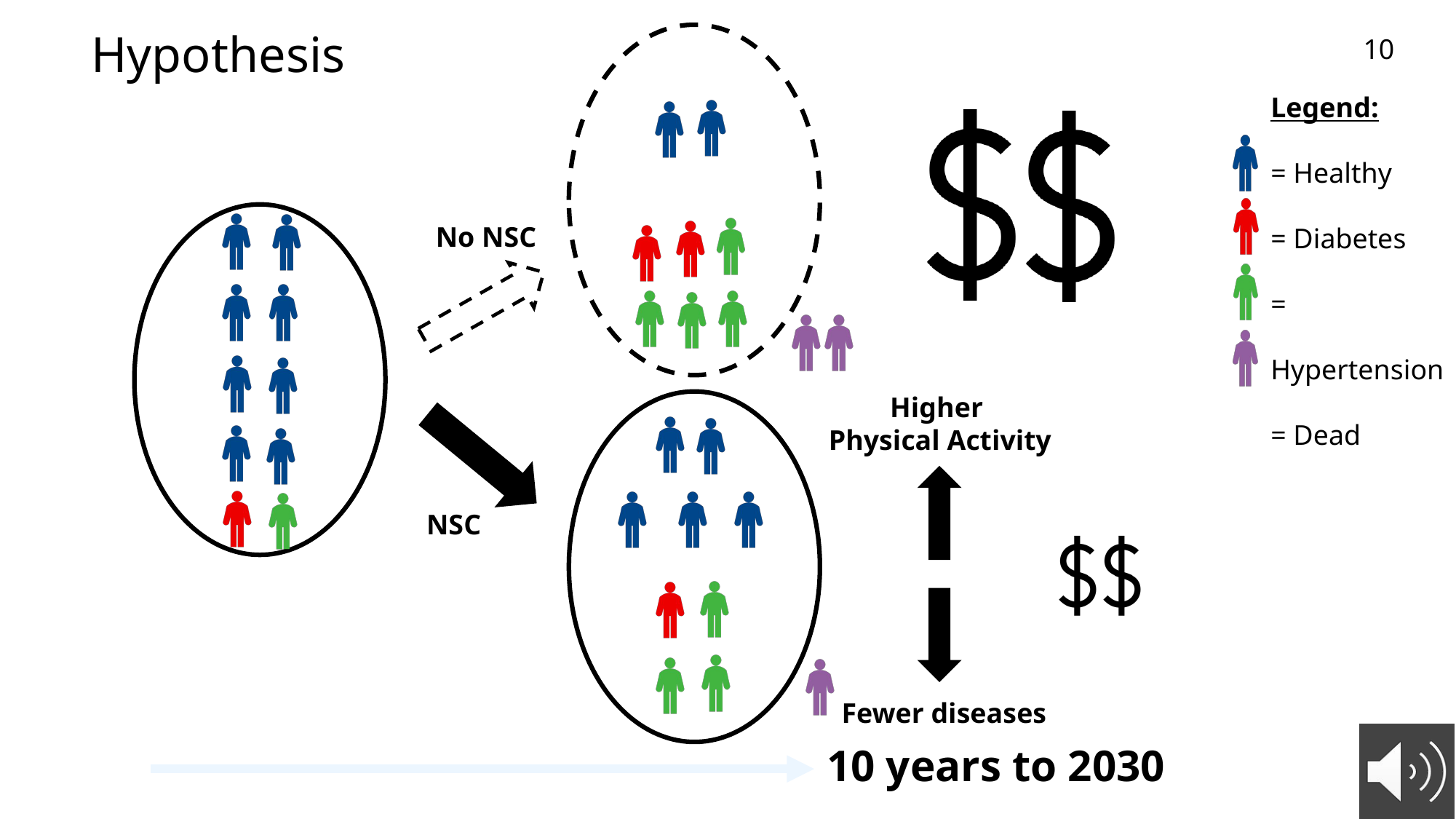

# Hypothesis
Legend:
= Healthy
= Diabetes
= Hypertension
= Dead
No NSC
Higher
Physical Activity
NSC
Fewer diseases
10 years to 2030

## Slide 11
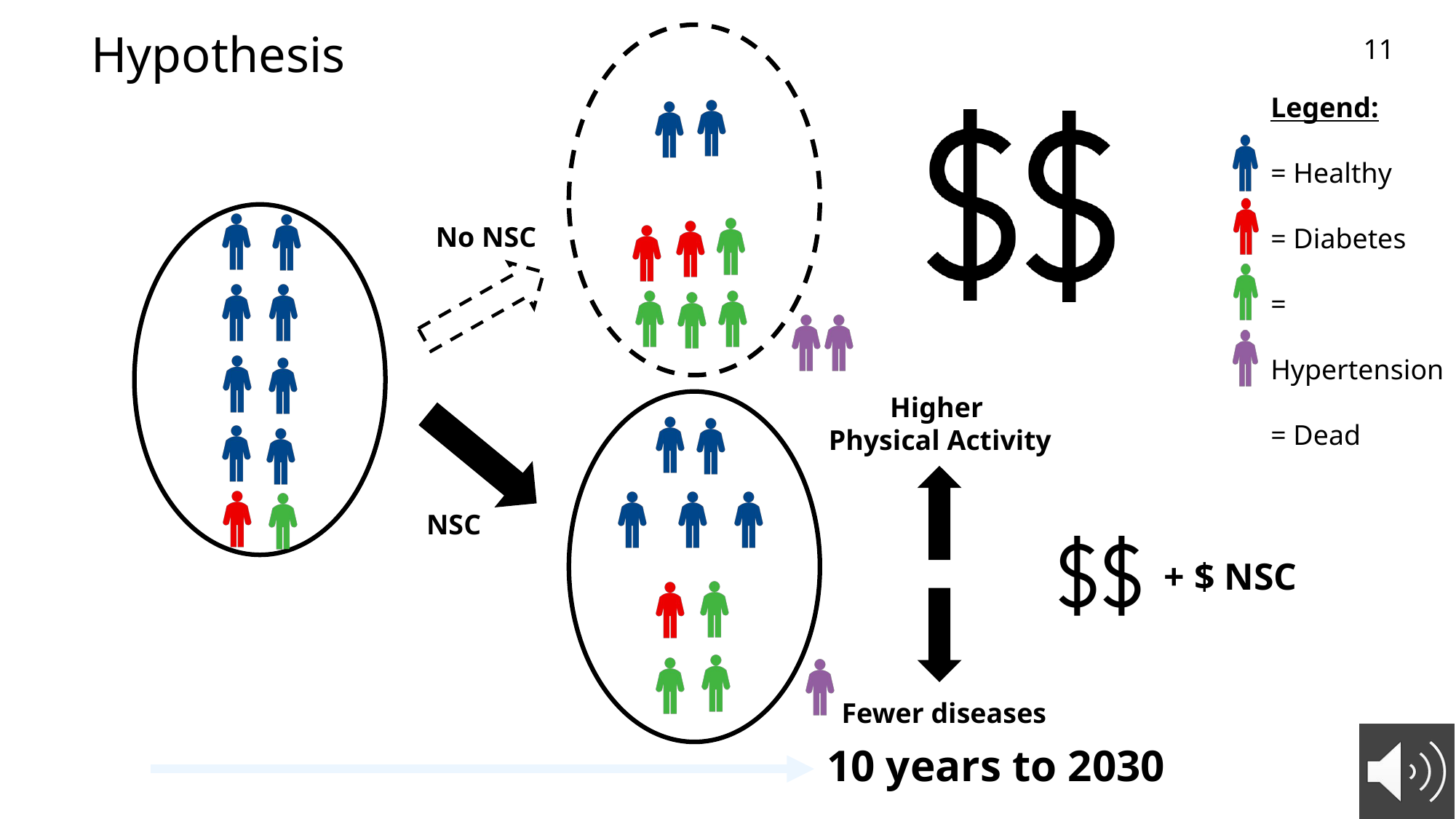

# Hypothesis
Legend:
= Healthy
= Diabetes
= Hypertension
= Dead
No NSC
Higher
Physical Activity
NSC
+ $ NSC
Fewer diseases
10 years to 2030

## Slide 12
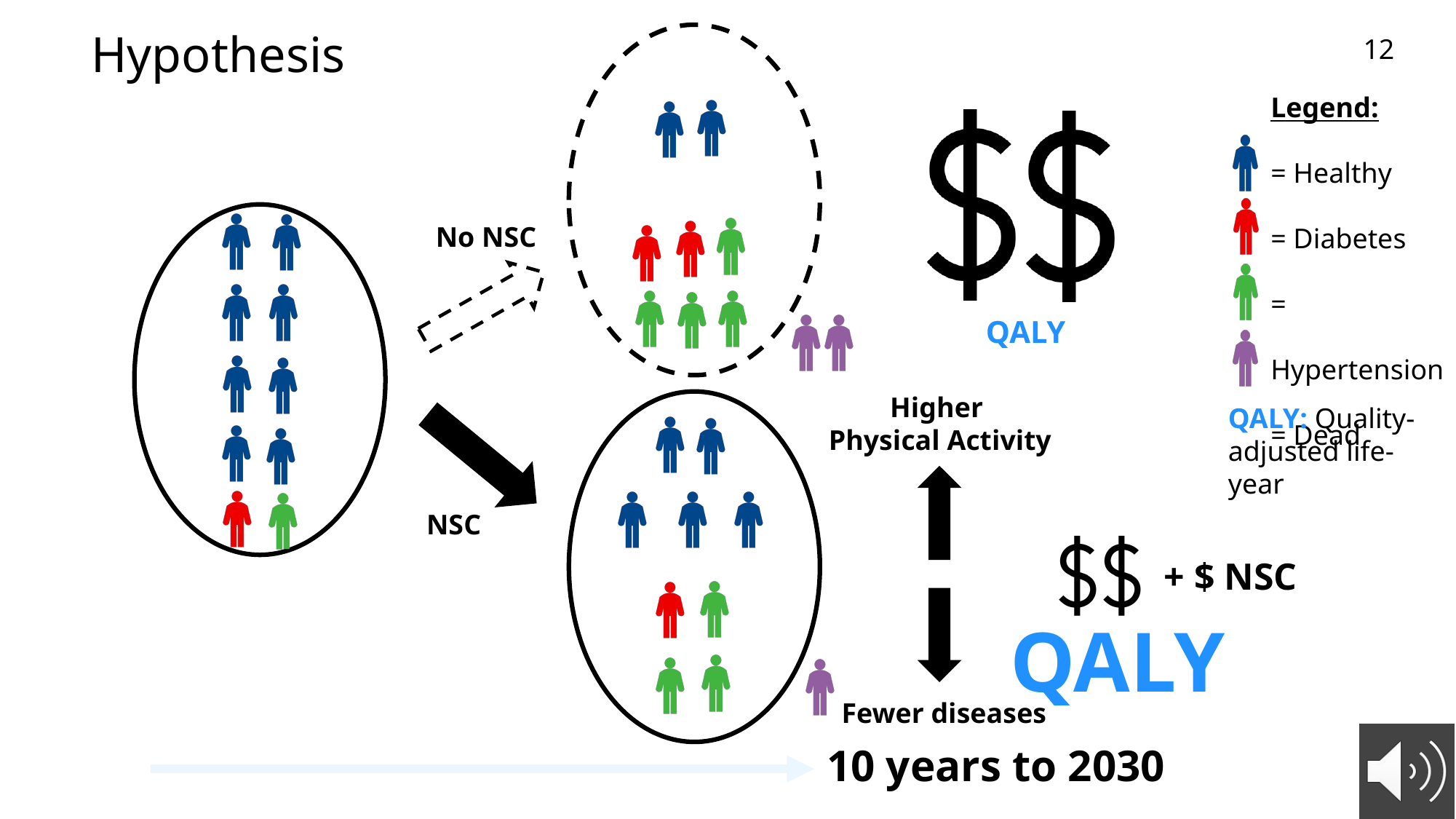

# Hypothesis
Legend:
= Healthy
= Diabetes
= Hypertension
= Dead
No NSC
QALY
Higher
Physical Activity
QALY: Quality-adjusted life-year
NSC
+ $ NSC
QALY
Fewer diseases
10 years to 2030

## Slide 13
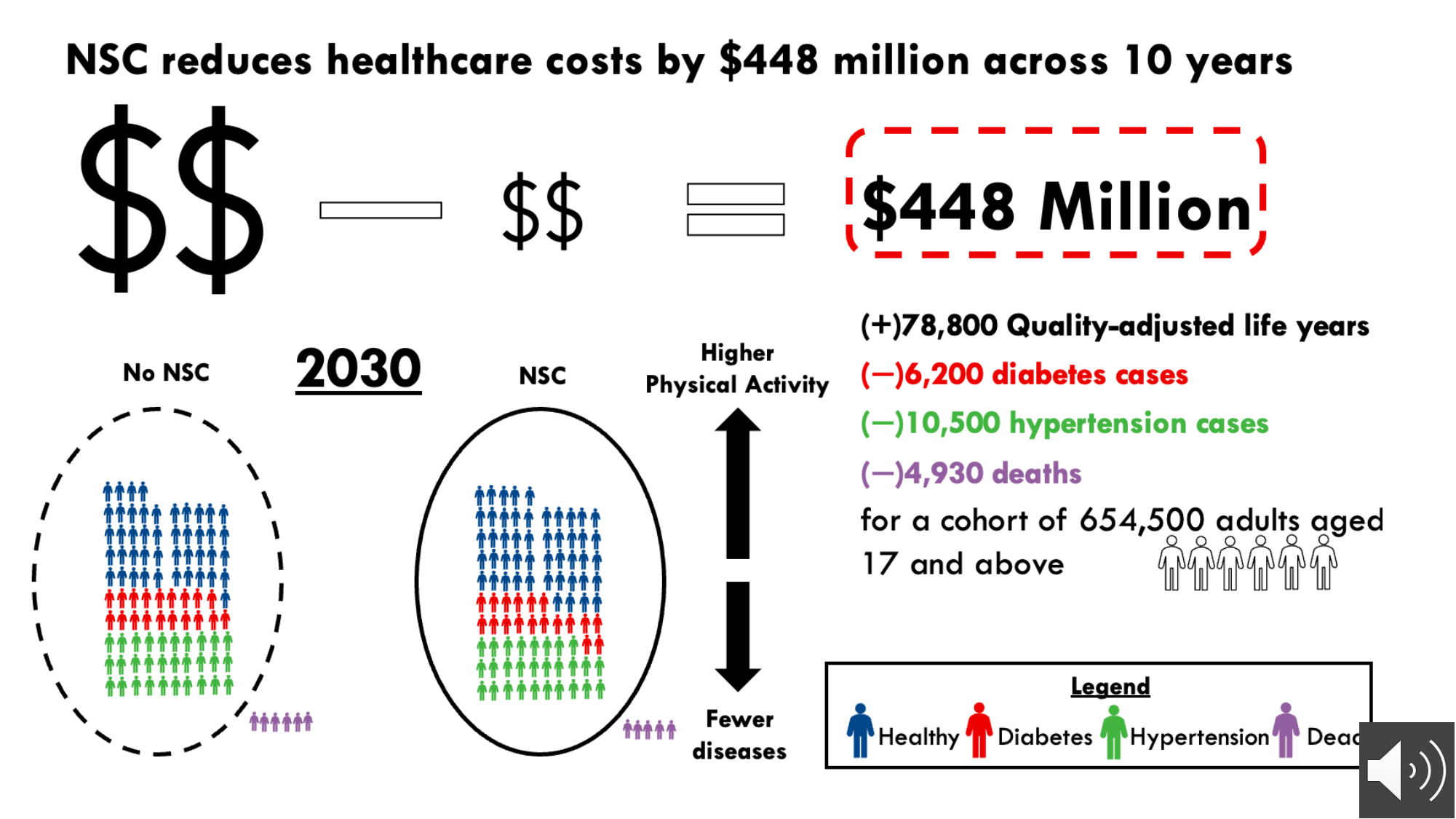

Supplement: Multimedia Appendix 2 [file publichealth-v10-e46178-s002.pptx]
